# Predicting Lung Cancer Survival to the Future: Population-Based Cancer Survival Modeling Study

**DOI:** 10.2196/46737

**Published:** 2024-05-31

**Authors:** Fan-Tsui Meng, Jing-Rong Jhuang, Yan-Teng Peng, Chun-Ju Chiang, Ya-Wen Yang, Chi-Yen Huang, Kuo-Ping Huang, Wen-Chung Lee

**Affiliations:** 1 Institute of Epidemiology and Preventive Medicine College of Public Health National Taiwan University Taipei Taiwan; 2 Parexel International Company Limited Taipei Taiwan; 3 Institute of Statistical Science Academia Sinica Taipei Taiwan; 4 Taiwan Cancer Registry Taipei Taiwan; 5 Health Promotion Administration Ministry of Health and Welfare Taipei Taiwan; 6 Institute of Health Data Analytics and Statistics College of Public Health National Taiwan University Taipei Taiwan

**Keywords:** lung cancer, survival, survivorship-period-cohort model, prediction, prognosis, early diagnosis, lung cancer screening, survival trend, population-based, population health, public health, surveillance, low-dose computed tomography

## Abstract

**Background:**

Lung cancer remains the leading cause of cancer-related mortality globally, with late diagnoses often resulting in poor prognosis. In response, the Lung Ambition Alliance aims to double the 5-year survival rate by 2025.

**Objective:**

Using the Taiwan Cancer Registry, this study uses the survivorship-period-cohort model to assess the feasibility of achieving this goal by predicting future survival rates of patients with lung cancer in Taiwan.

**Methods:**

This retrospective study analyzed data from 205,104 patients with lung cancer registered between 1997 and 2018. Survival rates were calculated using the survivorship-period-cohort model, focusing on 1-year interval survival rates and extrapolating to predict 5-year outcomes for diagnoses up to 2020, as viewed from 2025. Model validation involved comparing predicted rates with actual data using symmetric mean absolute percentage error.

**Results:**

The study identified notable improvements in survival rates beginning in 2004, with the predicted 5-year survival rate for 2020 reaching 38.7%, marking a considerable increase from the most recent available data of 23.8% for patients diagnosed in 2013. Subgroup analysis revealed varied survival improvements across different demographics and histological types. Predictions based on current trends indicate that achieving the Lung Ambition Alliance’s goal could be within reach.

**Conclusions:**

The analysis demonstrates notable improvements in lung cancer survival rates in Taiwan, driven by the adoption of low-dose computed tomography screening, alongside advances in diagnostic technologies and treatment strategies. While the ambitious target set by the Lung Ambition Alliance appears achievable, ongoing advancements in medical technology and health policies will be crucial. The study underscores the potential impact of continued enhancements in lung cancer management and the importance of strategic health interventions to further improve survival outcomes.

## Introduction

Lung cancer is the leading cause of cancer-related deaths worldwide. Approximately 1.8 million people worldwide died of lung cancer in 2020, accounting for 18% of all cancer deaths [1]. The disease typically lacks noticeable symptoms in its early stages, leading to late diagnoses in many patients. By the time symptoms such as coughing, shortness of breath, weakness, and chest pain manifest, the prognosis is often poor [2]. Lung cancer survival rates have improved slowly compared with other types of cancer [2-4]. The 5-year survival rate for lung cancer was approximately 10%-20% and is among the lowest of all types of cancer [5,6]. In Taiwan, lung cancer has been the leading cause of cancer death since 2010, with a 5-year survival rate of 25% [7].

Enhanced screening and early diagnosis are critical for improving prognosis and reducing mortality rates associated with lung cancer. The adoption of various diagnostic techniques, including blood tests and computed tomography scans, has significantly improved the accuracy and precision of lung cancer diagnoses [8-11]. Moreover, the advancement of noninvasive diagnostic methods has expedited the time required to reach a diagnosis [12-16]. In Taiwan, the implementation of low-dose computed tomography (LDCT) for lung cancer screening has been particularly effective. Data indicate a notable improvement in lung cancer survival rates across all patient groups from 2010 to 2016 [17].

In July 2019, the Lung Ambition Alliance, an international coalition dedicated to lung cancer control, announced its goal to double the 5-year survival rate for lung cancer by 2025 [18]. For this study, we used data from the Taiwan Cancer Registry, a national population-based registry known for its high-quality data [19,20]. We applied the survivorship-period-cohort (SPC) model, a recently developed method for nowcasting and forecasting cancer survival rates [21], to predict the future survival of patients with lung cancer in Taiwan. This analysis aims to assess the feasibility of achieving the coalition’s ambitious goal.

## Methods

### Case Definitions

Lung cancer data were obtained from the Taiwan Cancer Registry. Lung cancer cases were defined per the *International Classification of Disease for Oncology, Field Trial Edition* (ICD-O-FT) code 162 and *International Classification of Disease for Oncology, Third Edition* (ICD-O-3) codes C33 and C34. This study included patients diagnosed with lung cancer between July 1, 1997, and June 30, 2018. We linked patients to death registration data and tracked the date of death to December 31, 2018.

The year of diagnosis was defined as the middle of that year to the middle of the following year. For a given year of diagnosis, the number of survivors in the first year after diagnosis was the number of patients who did not die by the end of the following year. The first-year survival rate was calculated by dividing the number of patients who survived the first year after diagnosis by the number of patients in that year. Patients who survived the first year of diagnosis were followed up for another year, and the number of patients who did not die was the number of survivors in the second year after diagnosis. The second-year survival rate was calculated by dividing the number of survivors in the second year after diagnosis by the number of survivors in the first year. We calculated the survival rates for the third, fourth, and fifth years after diagnosis by using the same approach.

### Definitions of Survival Rates

**Figure 1:**
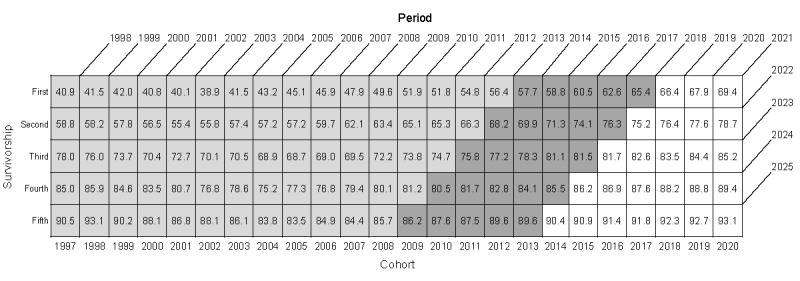
The unit of analysis for this study. The values in the cells are the 1-year interval survival rates (%) of lung cancer in Taiwan (shaded cells: observed values and unshaded cells: projected values from the survivorship-period-cohort model).

The unit of analysis in this study was the 1-year interval survival rate, defined as the probability that a patient with cancer currently alive will survive for at least 1 more year. The 1-year interval survival rates for patients with lung cancer based on the year after diagnosis (survivorship), calendar year (period), and year of diagnosis (cohort) are presented in Figure 1 (a total of 95 shaded cells with observed values). The 5-year survival rate, calculated using the cohort approach, is the product of the five 1-year interval (first to fifth years) survival rates for patients diagnosed in the same year. We extrapolated the 1-year interval survival rate to the 2020 year of diagnosis (25 unshaded cells in Figure 1). In the cohort approach, the 5-year survival rate for calendar year 2025 is the 5-year survival rate for patients diagnosed in 2020.

### SPC Model

This study used the SPC model proposed by Peng et al [21] to predict survival rates. The independent variables were the year after diagnosis (survivorship), calendar year (period), and year of diagnosis (cohort). These 3 variables are perfectly collinear (cohort + survivorship = period), resulting in a nonidentifiability problem. The nonidentifiability problem leads to infinite possibilities for parameter estimation. However, this does not prevent the prediction of the 1-year interval survival rate because all possible parameter estimates yield the same fitted value. The overall effects of period and cohort were divided into “slopes” and “curvatures” for extrapolation. We projected that the future period and cohort slopes would be identical to those observed in the empirical data and used the restricted cubic spline method to fit the period and cohort curvatures of the empirical data to extrapolate into the future. A more detailed description of the SPC model can be found in Peng et al [21].

### Training and Validation

We used the 1-year interval survival rates from 1998 to 2013 as the training data set (70 lightly shaded cells in Figure 1) and the 1-year interval survival rates from 2014 to 2018 as the validation data set (25 darkly shaded cells in Figure 1) to calculate the symmetric mean absolute percentage error for the SPC model. We used all observed data (95 shaded cells in Figure 1) to predict the 1-year interval survival rates. We multiplied the 5 predicted 1-year interval (first to fifth year after diagnosis) survival rates (patients diagnosed between 2018 and 2020) or the partially observed and partially predicted 1-year interval survival rates (patients diagnosed between 2014 and 2017) for patients diagnosed in the same year to obtain the expected 5-year survival rate.

### Uncertainty in Prediction

We used the bootstrap method to calculate CIs for the survival rates. We performed 10,000 bootstrap resampling and used the interval between the 2.5th and 97.5th percentiles as the 95% CI for the survival rates.

### Data Analysis Software Used

All analyses were performed using SAS (version 9.4; SAS Institute Inc) and R (version 4.3.1; R Foundation for Statistical Computing).

### Ethical Considerations

The study used deidentified aggregate data exclusively without access to individual records. Ethical approval was granted by the National Taiwan University Research Ethics Committee (NTU-REC 202101HM030) and the Data Release Review Board of the Health Promotion Administration, Ministry of Health and Welfare in Taiwan. All procedures were conducted in compliance with applicable guidelines and regulations. Additionally, the requirement for informed consent was waived by the National Taiwan University Research Ethics Committee due to the absence of personal information and the use of secondary data in the study.

## Results

### Characteristics of the Study Population

This study analyzed data from 205,104 patients with lung cancer, of whom 128,928 were men and 76,176 were women (Table 1). In total, 8236 (56.7%) patients were older than 65 years of age. The number of newly diagnosed cases significantly increased from 5762 in 1997 to 14,529 in 2017. Among all histological types, lung adenocarcinoma was the most prevalent in Taiwan, with its proportion rising sharply from 2202 (38.2%) in 1997 to 10,223 (70.4%) in 2017. Lung squamous cell carcinoma was the second most common type, with its proportion decreasing from 1448 (25.1%) in 1997 to 1856 (12.8%) in 2017. Regarding stage distribution, most patients with lung cancer in Taiwan were diagnosed at stages III and IV; however, this proportion gradually decreased from 5933 (83.8%) in 1997 to 8340 (66.8%) in 2017. Conversely, the proportion of stage I diagnoses increased from 927 (13.1%) in 1997 to 3659 (29.3%) in 2017.

**Table 1:** Patients with lung cancer diagnosed in Taiwan from 1997 to 2017, grouped by sex, age, histological type, and stage.

Year	Sex, n (%)			Age (years), n (%)					Histological type, n (%)					Stage, n (%)					Total
	Male	Female	<55		55-64	65-74	≥75	ADENO^a^		SCC^b^	SCLC^c^	Others	I		II	III	IV		
1997	4041 (70.1)	1721 (29.9)	850 (14.8)		1186 (20.6)	2248 (39)	1478 (25.7)	2202 (38.2)		1448 (25.1)	503 (8.7)	1609 (27.9)	—^d^		—	—	—	5762	
1998	4425 (63.3)	1963 (30.7)	926 (14.5)		1253 (19.6)	2473 (38.7)	1736 (27.2)	2505 (39.2)		1589 (24.9)	550 (8.6)	1744 (27.3)	—		—	—	—	6388	
1999	4681 (68.9)	2114 (31.1)	1015 (14.9)		1249 (18.4)	2599 (38.2)	1932 (28.4)	2790 (41.1)		1667 (24.5)	607 (8.9)	1731 (25.5)	—		—	—	—	6795	
2000	4634 (69.7)	2013 (30.3)	972 (14.6)		1197 (18)	2509 (37.7)	1969 (29.7)	2789 (42)		1637 (24.6)	625 (9.4)	1596 (24)	—		—	—	—	6647	
2001	4808 (68)	2265 (32)	1124 (15.9)		1292 (18.3)	2444 (34.6)	2213 (31.3)	3136 (44.3)		1594 (22.5)	627 (8.9)	1716 (24.3)	—		—	—	—	7073	
2002	4788 (67.4)	2316 (32.6)	1134 (16)		1260 (17.7)	2427 (34.2)	2283 (32.1)	3087 (43.5)		1536 (22)	632 (8.9)	1822 (25.6)	—		—	—	—	7104	
2003	5468 (66.8)	2720 (33.2)	1288 (15.7)		1421 (17.4)	2677 (32.7)	2802 (34.2)	3688 (45)		1735 (21.2)	776 (9.5)	1989 (24.3)	—		—	—	—	8188	
2004	5746 (67.8)	2727 (32.2)	1315 (15.5)		1470 (17.3)	2619 (30.9)	3069 (36.2)	3811 (45)		1833 (21.6)	767 (9.1)	2062 (24.3)	—		—	—	–—	8473	
2005	5627 (65.6)	2954 (34.4)	1453 (16.9)		1491 (17.4)	2545 (29.7)	3092 (36)	4205 (49)		1759 (20.5)	751 (8.8)	1866 (21.7)	—		—	—	–—	8581	
2006	6064 (65.8)	3150 (34.2)	1468 (15.9)		1621 (17.6)	2644 (28.7)	3481 (37.8)	4664 (50.6)		1825 (19.8)	844 (9.2)	1881 (20.4)	—		—	—	–—	9214	
2007	6249 (64.6)	3424 (35.4)	1627 (16.8)		1749 (18.1)	2796 (28.9)	3501 (36.2)	5058 (52.3)		1801 (18.6)	856 (8.8)	1958 (20.2)	927 (13.1)		223 (3.1)	1840 (26)	4093 (57.8)	9673	
2008	6625 (64.6)	3633 (35.4)	1600 (15.6)		1948 (19)	2873 (28)	3837 (37.4)	5578 (54.4)		1813 (17.7)	981 (9.6)	1886 (18.4)	959 (13.3)		255 (3.5)	1843 (25.6)	4148 (57.6)	10,258	
2009	6555 (62.7)	3902 (37.3)	1638 (15.7)		2177 (20.8)	2789 (26.7)	3853 (36.8)	5945 (56.9)		1794 (17.2)	877 (8.4)	1841 (17.6)	1390 (15)		267 (2.9)	2290 (24.6)	5345 (57.5)	10,457	
2010	6824 (63.2)	3965 (36.8)	1767 (16.4)		2273 (21.1)	2787 (25.8)	3962 (36.7)	6174 (57.2)		1976 (18.3)	901 (8.4)	1738 (16.1)	1298 (14.1)		462 (5)	1731 (18.8)	5719 (62.1)	10,789	
2011	7071 (61.4)	4451 (38.6)	1908 (16.6)		2570 (22.3)	2901 (25.2)	4143 (36)	7011 (60.8)		1925 (16.7)	876 (7.6)	1710 (14.8)	1324 (14.4)		422 (4.6)	1662 (18.1)	5797 (63)	11,522	
2012	6971 (60.8)	4499 (39.2)	1871 (16.3)		2761 (24.1)	2908 (25.4)	3930 (34.3)	7162 (62.4)		1871 (16.3)	866 (7.6)	1571 (13.7)	1744 (17.6)		435 (4.4)	1700 (17.2)	6009 (60.8)	11,470	
2013	7312 (59.7)	4944 (40.3)	2082 (17)		3012 (24.6)	3113 (25.4)	4049 (33)	7851 (64.1)		1904 (15.5)	938 (7.7)	1563 (12.8)	1966 (19.5)		448 (4.4)	1657 (16.4)	6015 (59.6)	12,256	
2014	7380 (58.5)	5230 (41.5)	2067 (16.4)		3192 (25.3)	3192 (25.3)	4159 (33)	8156 (64.7)		1920 (15.2)	943 (7.5)	1591 (12.6)	2174 (20.3)		511 (4.8)	1658 (15.5)	6355 (59.4)	12,610	
2015	7715 (57.7)	5660 (42.3)	2181 (16.3)		3432 (25.7)	3473 (26)	4289 (32.1)	8920 (66.7)		1906 (14.3)	959 (7.2)	1590 (11.9)	2647 (23.5)		500 (4.4)	1700 (15.1)	6395 (56.9)	13,375	
2016	7889 (57.4)	5903 (42.8)	2250 (16.3)		3534 (25.6)	3724 (27)	4284 (31.1)	9333 (67.7)		1911 (13.9)	944 (7.2)	1554 (11.3)	3088 (26.4)		513 (4.4)	1670 (14.3)	6433 (55)	13,792	
2017	8008 (55.1)	6521 (44.9)	2451 (16.9)		3842 (26.4)	3950 (27.2)	4286 (29.5)	10,223 (70.4)		1856 (12.8)	984 (6.8)	1466 (10.1)	3659 (29.3)		499 (4)	1784 (14.3)	6556 (52.5)	14,529	

^a^ADENO: adenocarcinoma.

^b^SCC: squamous cell carcinoma.

^c^SCLC: small cell lung cancer.

^d^Not available.

### Trends in Survival Rates

**Figure 2:**
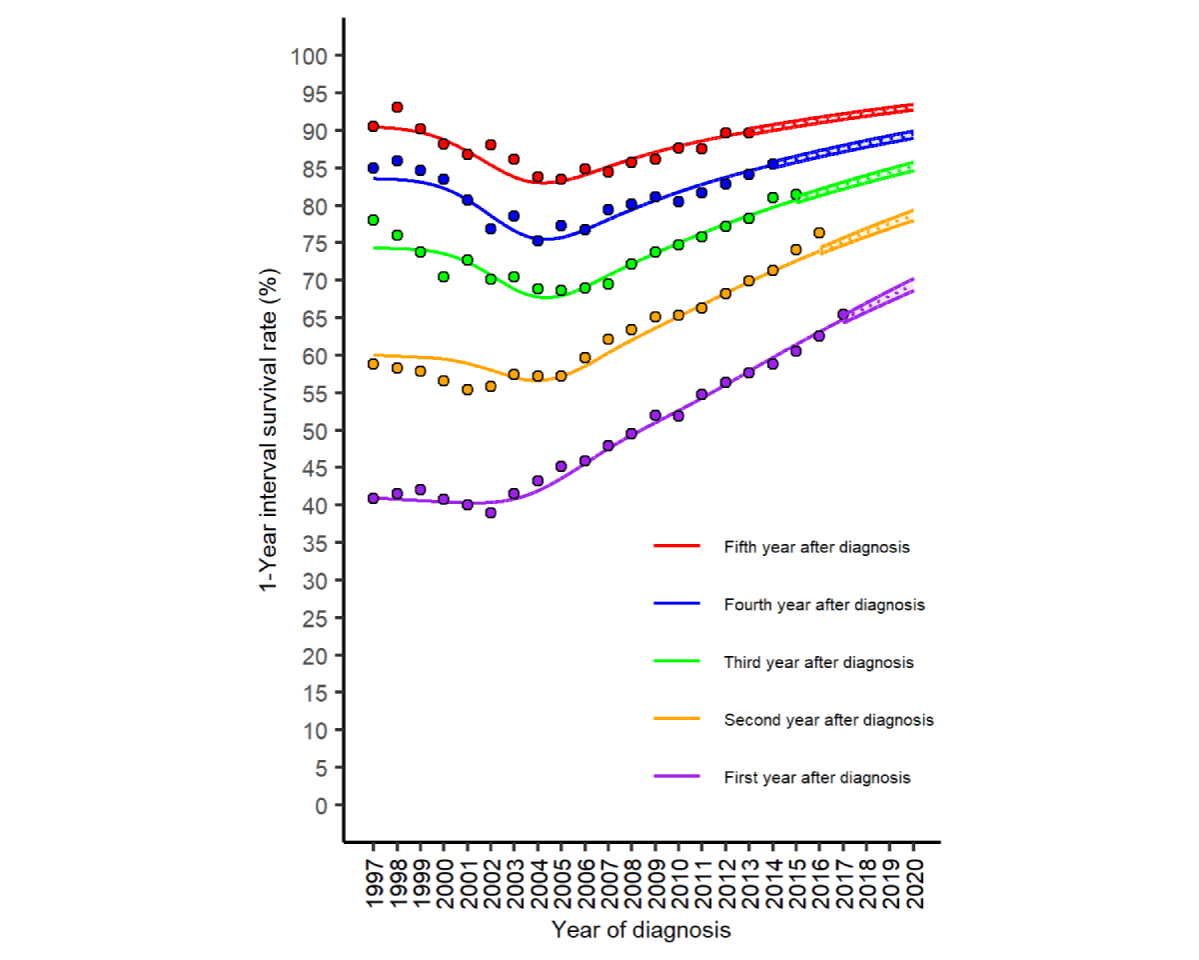
The 1-year interval survival rates (%) of lung cancer in Taiwan (dots: observed values; solid lines: fitted values from the survivorship-period-cohort model; dotted lines: projected values from the survivorship-period-cohort model; and color-shaded regions: 95% bootstrap CI).

Figure 2 displays the 1-year interval survival rates from the first to the fifth year after lung cancer diagnosis in Taiwan. The 1-year interval survival rates from the first to the fifth year had the same turning point at the diagnosis year of 2004: falling before this year and rising subsequently. The fastest increase in the 1-year interval survival rate is the first year after diagnosis. In the year of diagnosis 2020, the 1-year interval survival rates are projected to reach 69.4%, 78.7%, 85.2%, 89.4%, and 93.1% in the first, second, third, fourth, and fifth years after diagnosis, respectively.

**Figure 3:**
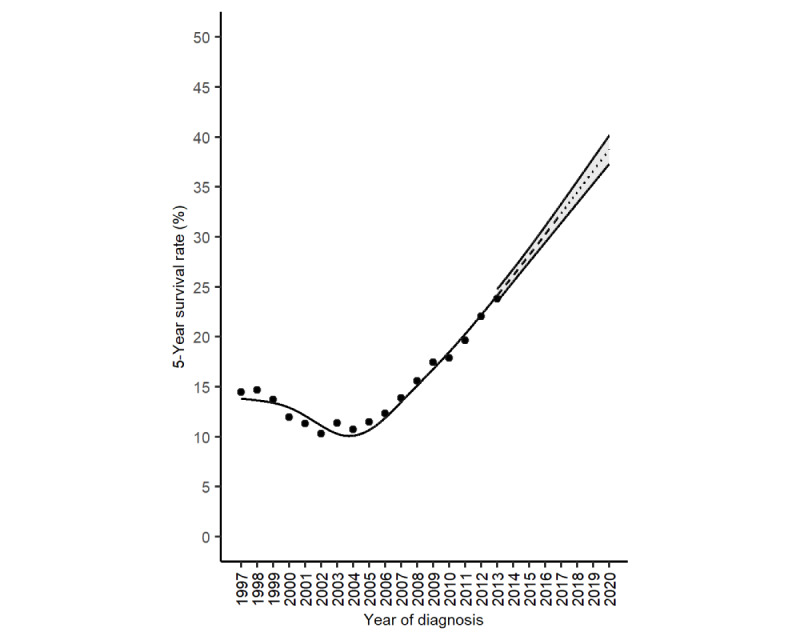
The 5-year survival rates (%) of lung cancer in Taiwan (dots: observed values; solid line: product of 5 observed 1-year interval survival rates from the first to fifth years after diagnosis for patients in the same diagnosis year, 1997-2013; dashed line: product of 5 partially observed and partially predicted 1-year interval survival rates from the first to fifth years after diagnosis for patients in the same diagnosis year, 2014-2017; dotted line: product of 5 predicted 1-year survival rates from the first to fifth years after diagnosis for patients in the same diagnosis year, 2018 to 2020; and shaded region: 95% bootstrap CI).

Figure 3 displays the 5-year survival rate of lung cancer in Taiwan. The most recent 5-year survival rate that could be computed using the cohort approach was the 5-year survival rate for patients diagnosed in 2013 (23.8%). We used the SPC model (symmetric mean absolute percentage error=0.72%) to predict a 5-year survival rate of 38.7% for the year of diagnosis 2020. The absolute increase in the 5-year survival rate of lung cancer in Taiwan from the year of diagnosis 2013 to 2020 was 38.7% – 23.8% = 14.9%, and the fold increase was 38.7%/23.8% = 1.6-fold.

### Trends in Relative Survival Rates

This study also assessed the relative survival rate of patients with lung cancer (relative to the survival rate of the age-, sex-, and calendar year–matched population in Taiwan) [21,22]. Both the relative survival rates (Figures S1 and S2 in Multimedia Appendix 1) and the absolute survival rates (Figures 2 and 3) exhibited similar increasing trends. The absolute increase in the 5-year relative survival rate of lung cancer in Taiwan from year of diagnosis 2013 to 2020 was 44.6% – 27.9% = 16.7%, and the fold increase was 44.9%/27.9% = 1.6-fold.

### Subgroup Analysis

In addition, we predicted the future survival rates of patients with lung cancer on the basis of sex, age, and histological type (Figures S3-S15 in Multimedia Appendix 1). The absolute increases (fold increases) in the 5-year survival rates of patients with lung cancer are as follows (Table S1 in Multimedia Appendix 1): men 9.8% (1.6-fold), women 19% (1.6-fold), younger than 55 years 23.4% (1.6-fold), 55 to 64 years 16.8% (1.5-fold), 65 to 74 years 15.5% (1.6-fold), older than 75 years 4.9% (1.5-fold), lung adenocarcinoma 18.6% (1.6-fold), lung squamous cell carcinoma 2.4% (1.2-fold), small-cell lung cancer 1% (1.2-fold), and other histological types of lung cancer 2.6% (1.2-fold).

### Weighted Average Predictions Across Patient Strata

**Figure 4:**
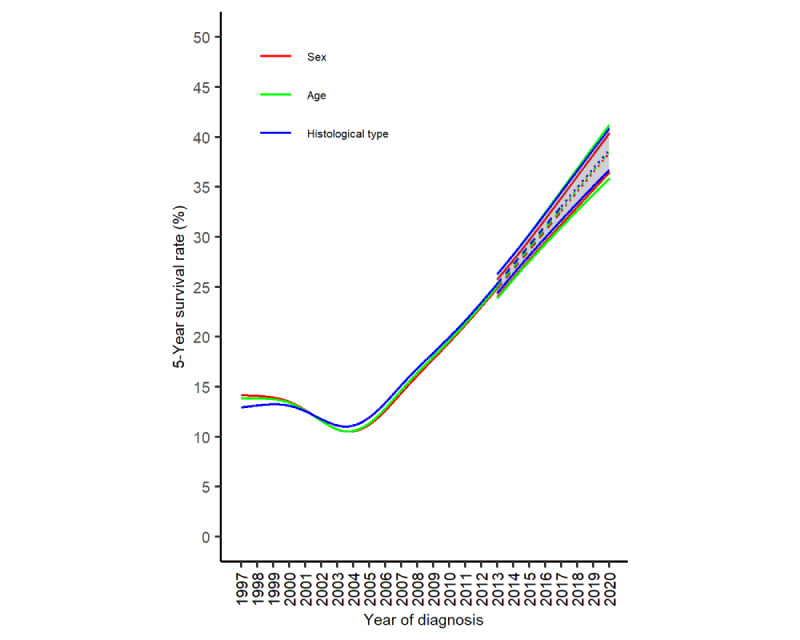
The 5-year survival rates (%) of lung cancer in Taiwan, weighted averaged by sex, age, and histological type of the number of patients diagnosed in 2017 (solid line: product of 5 observed 1-year interval survival rates from the first to fifth years after diagnosis for patients in the same diagnosis year, 1997-2013; dashed line: product of 5 partially observed and partially predicted 1-year interval survival rates from the first to fifth years after diagnosis for patients in the same diagnosis year, 2014-2017; dotted line: product of 5 predicted 1-year survival rates from the first to fifth years after diagnosis for patients in the same diagnosis year, 2018-2020; and color-shaded regions: 95% bootstrap CI).

We also made predictions by calculating the weighted average of stratum-specific future 5-year survival rates based on the number of patients in each stratum in the year of diagnosis 2017 (Table 1). The results (Figure 4) closely matched the future 5-year survival rates predicted for all patients with lung cancer (Figure 3).

## Discussion

### Principal Findings

This population-based cancer survival study demonstrates that the 5-year survival rate of patients with lung cancer in Taiwan decreased before 2004 but proceeded to increase afterward, corresponding to the trend of lung cancer mortality in Taiwan [23,24] and the trend of lung cancer survival in one hospital in Taiwan [24]. Patients with advanced lung cancer usually receive chemotherapy. Taiwan’s National Health Insurance has been reimbursing several targeted drugs for lung cancer treatment: gefitinib (since 2004), erlotinib (since 2007), afatinib (since 2014), and crizotinib (since 2015) [25]. These targeted drugs are mainly used in patients with lung adenocarcinoma, and their treatment effects are more favorable than chemotherapy [26-28]. Figure S15 and Table S1 in Multimedia Appendix 1 also reveal more favorable survival rates for patients with lung adenocarcinoma compared to other histological types. Taiwan’s National Health Insurance has also been reimbursing positron emission tomography for staging tumors since 2004 [29]. Previous studies have revealed that it is more accurate for staging tumors than computed tomography, enabling patients to receive appropriate treatment [30-32]. This may also be one reason the 5-year survival rate for lung cancer in Taiwan has increased since 2004. In addition, the Tobacco Hazards Prevention Act was implemented in 1997, and the smoking rate in Taiwan has dropped considerably. The incidences of small-cell lung cancer and lung squamous cell carcinoma, closely related to smoking, have also decreased yearly [33,34]. The reduced proportion of the poorer-survival histological types may contribute to the overall improvement in lung cancer survival.

Lung cancer screening can also help improve the survival rate of patients with lung cancer. The National Lung Screening Trial in the United States first demonstrated in 2011 that LDCT could effectively reduce lung cancer mortality [35]. The Lung Cancer Society in Taiwan has also conducted an LDCT screening study on nonsmoking lung cancer for high-risk populations [36]. LDCT can detect more early-stage patients and reduce lung cancer mortality compared to traditional chest radiography [35-38]. The proportion of patients with early-stage lung cancer diagnosed each year in Taiwan has been increasing since 2007, and the rate of increase has accelerated since 2011 (Figure S16 in Multimedia Appendix 1). Lung cancer survival rates have rapidly increased since 2007 when stages I to IV were combined (see solid line in Figures S17 and S18 in Multimedia Appendix 1). In addition, the survival rate in each stage exhibits an upward trend (Figures S19-S23 in Multimedia Appendix 1). We believe that positron emission tomography during this period allowed for more accurate staging [30-32] and improved survival rates at various stages. Targeted therapy may also contribute to the increased survival rate of patients with advanced disease [39-42].

We simulated a lung cancer survival trend in the event that screening did not take place (see dot-dashed line in Figures S17 and S18 in Multimedia Appendix 1). The trend was calculated as the yearly weighted averages of the stage-specific lung cancer survival rates, assuming the proportion of patients diagnosed in each stage remained constant at the 2007 level (Figure S16 in Multimedia Appendix 1). The difference between the factual trend (see solid line in Figures S17 and S18 in Multimedia Appendix 1) and the simulated counterfactual trend (see dot-dashed line in Figures S17 and S18 in Multimedia Appendix 1) represents the benefit of lung cancer screening, although this may also reflect the effect of lead time bias. The Health Promotion Administration of Taiwan started implementing free LDCT screening for lung cancer for the high-risk population [43] in July 2022 (before that, LDCT screening was paid for by the public at their own expense). As a result, we speculate that the 5-year survival rate of patients with lung cancer will continue to increase in the future.

The novelty and strength of this study lie in its scope—a nationwide, population-based cancer survival analysis involving more than 200,000 patients with lung cancer. Furthermore, it uses the state-of-the-art SPC model for macrosimulation of future trends. To the best of our knowledge, this is the first study of its kind. However, this study has several limitations. First, the forecasts do not account for the impacts of health policy implementations or medical advancements post-2018, which may result in an underestimation of the future 5-year survival rates for lung cancer in Taiwan. Second, as this is a population-based cancer survival modeling analysis, the absence of individual-level data hampers our ability to make causal inferences and precise personal predictions. Additionally, the factors contributing to the decline in lung cancer survival rates in Taiwan between 1997 and 2004 remain unclear.

### Conclusions

This study conducted a comprehensive analysis of lung cancer survival trends in Taiwan, using data from over 200,000 patients and using the SPC model to project future survival rates. Our findings indicate notable improvements in the 5-year survival rates, particularly noticeable from 2004 onward, attributed to advances in medical treatments and diagnostic methods, including the implementation of LDCT screening. The results suggest that the goal set by the Lung Ambition Alliance to double the 5-year survival rate by 2025 is ambitious yet potentially achievable within Taiwan. However, limitations such as the exclusion of post-2018 medical advancements and policy changes could affect the accuracy of our projections. This study highlights the critical role of continuous medical innovation and effective health policy implementation in achieving significant survival improvements for patients with lung cancer.

## Appendix

Multimedia Appendix 1 Stratification of overall and relative survival by sex, age, histology, and stage.

## Abbreviations

ICD-O-3: International Classification of Disease for Oncology, Third Edition
ICD-O-FT: International Classification of Disease for Oncology, Field Trial Edition
LDCT: low-dose computed tomography
SPC: survivorship-period-cohort

## Data Availability

The data sets generated and analyzed during this study are available from the corresponding author upon reasonable request.
