# Supplementary material for: Predicting Lung Cancer Survival to the Future: Population-Based Cancer Survival Modeling Study
Source: JMIR Public Health Surveill. 2024 May 31;10:e46737. doi: 10.2196/46737 (PMC11179019; doi:10.2196/46737)

Table S1. The five-year survival rate, absolute increases, and fold increases of lung cancer patients in Taiwan.

	5-year survival rate (%) at the year of diagnosis 2013 (A)	Predicted 5-year survival rate (%) for the year of diagnosis 2020 (B)	Absolute increases in 5-year survival rate (%) from the year of diagnosis 2013 to 2020 (B－A)	Fold increases in 5-year survival rate (%) from the year of diagnosis 2013 to 2020 (B/A)
Total	23.8	38.7	14.9	1.6
Sex				
Men	17.7	27.5	9.8	1.6
Women	32.8	51.8	19.0	1.6
Age group				
under 55	36.6	60.1	23.4	1.6
55 to 64	32.6	49.4	16.8	1.5
65 to 74	25.0	40.4	15.5	1.6
over 75	9.7	14.6	4.9	1.5
Histological type				
Adenocarcinoma	30.7	49.4	18.6	1.6
Squamous cell carcinoma	13.1	15.5	2.4	1.2
Small cell lung cancer	4.9	5.9	1.0	1.2
Others	13.2	15.9	2.6	1.2
Stage				
I	81.0	93.9	12.9	1.2
II	48.9	67.4	18.5	1.4
III	24.5	37.3	12.8	1.5
IV	7.9	16.9	9.0	2.1

Figure S1. The 1-year interval relative survival rates (%) of lung cancer in Taiwan (dots: observed values; solid lines: fitted values from the survivorship-period-cohort model; dotted lines: projected values from the survivorship-period-cohort model; color-shaded regions: 95% bootstrap confidence intervals).

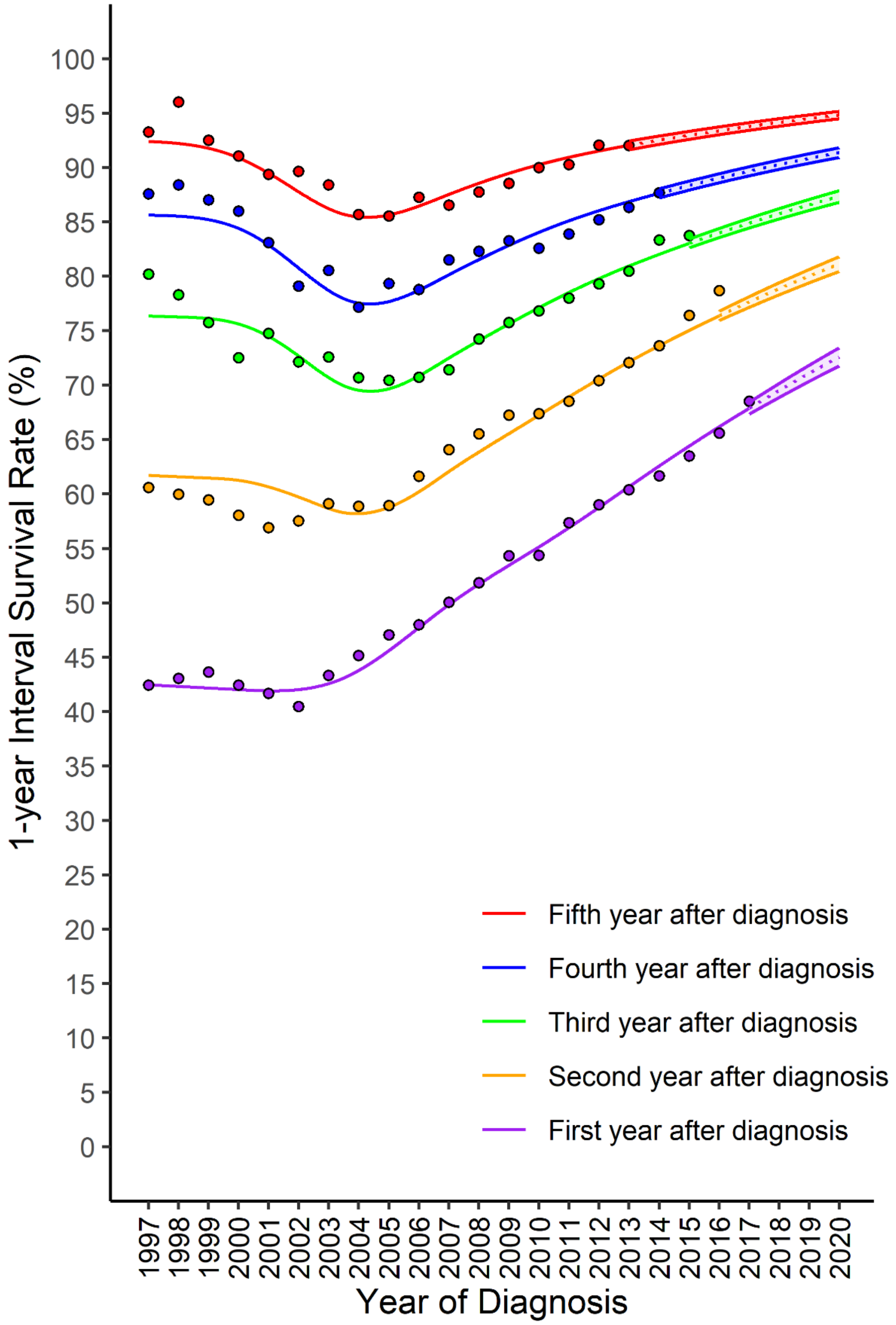

Figure S2. The 5-year relative survival rates (%) of lung cancer in Taiwan (dots: observed values; solid line: product of five observed 1-year interval relative survival rates from the first to fifth years after diagnosis for patients in the same diagnosis year, 1997-2013; dashed line: product of five partially observed and partially predicted 1-year interval relative survival rates from the first to fifth years after diagnosis for patients in the same diagnosis year, 2014-2017; dotted line: product of five predicted 1-year survival relative rates from the first to fifth years after diagnosis for patients in the same diagnosis year, 2018 to 2020; shaded region: 95% bootstrap confidence interval).

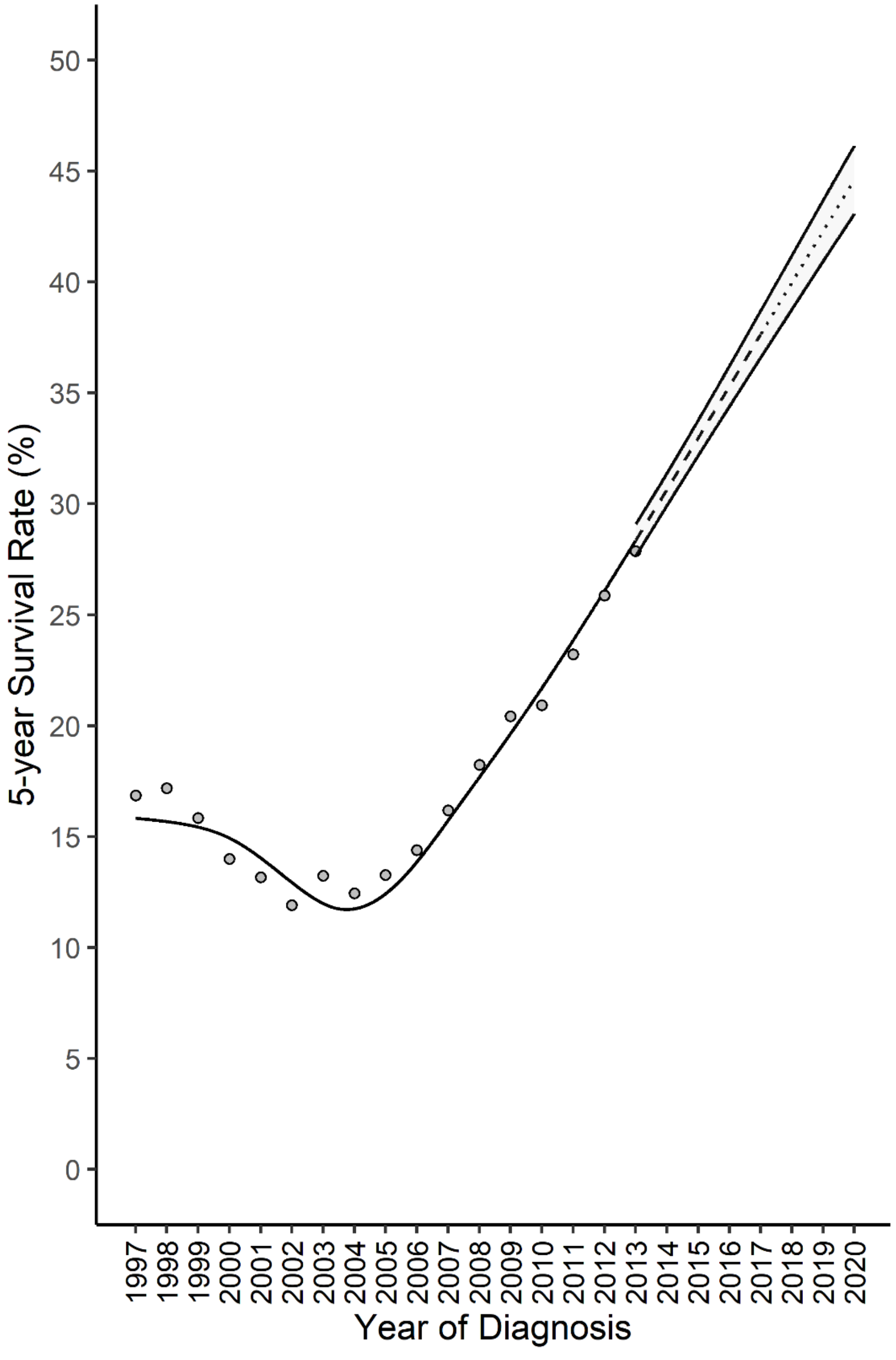

Figure S3. The 1-year interval survival rates (%) for male with lung cancer in Taiwan (dots: observed values; solid lines: fitted values from the survivorship-period-cohort model; dotted lines: projected values from the survivorship-period-cohort model; color-shaded regions: 95% bootstrap confidence intervals).

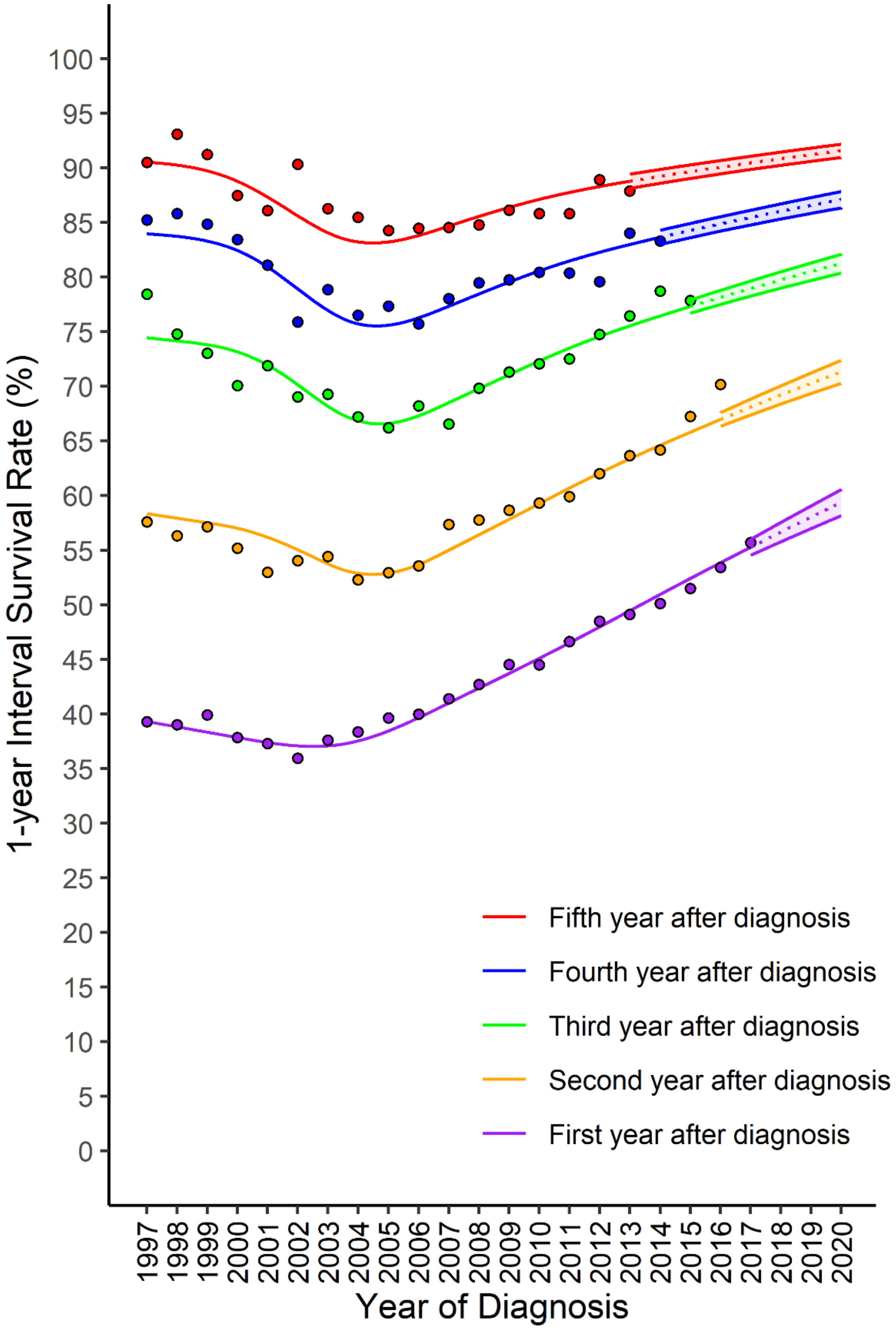

Figure S4. The 1-year interval survival rates (%) for female with lung cancer in Taiwan (dots: observed values; solid lines: fitted values from the survivorship-period-cohort model; dotted lines: projected values from the survivorship-period-cohort model; color-shaded regions: 95% bootstrap confidence intervals).

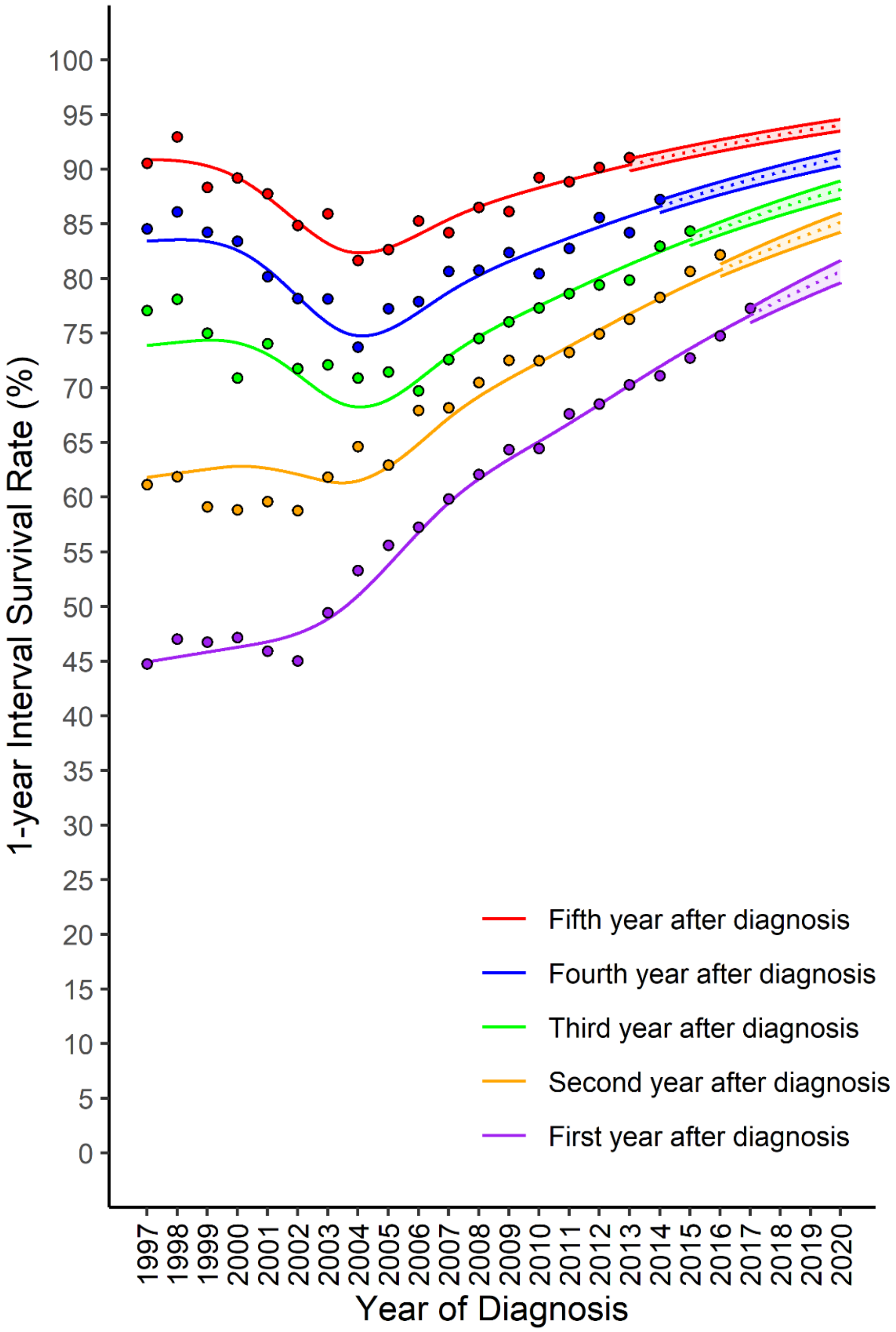

Figure S5. The 5-year survival rates (%) of lung cancer by sex in Taiwan (dots: observed values; solid line: product of five observed 1-year interval survival rates from the first to fifth years after diagnosis for patients in the same diagnosis year, 1997-2013; dashed line: product of five partially observed and partially predicted 1-year interval survival rates from the first to fifth years after diagnosis for patients in the same diagnosis year, 2014-2017; dotted line: product of five predicted 1-year survival rates from the first to fifth years after diagnosis for patients in the same diagnosis year, 2018 to 2020; shaded region: 95% bootstrap confidence interval).

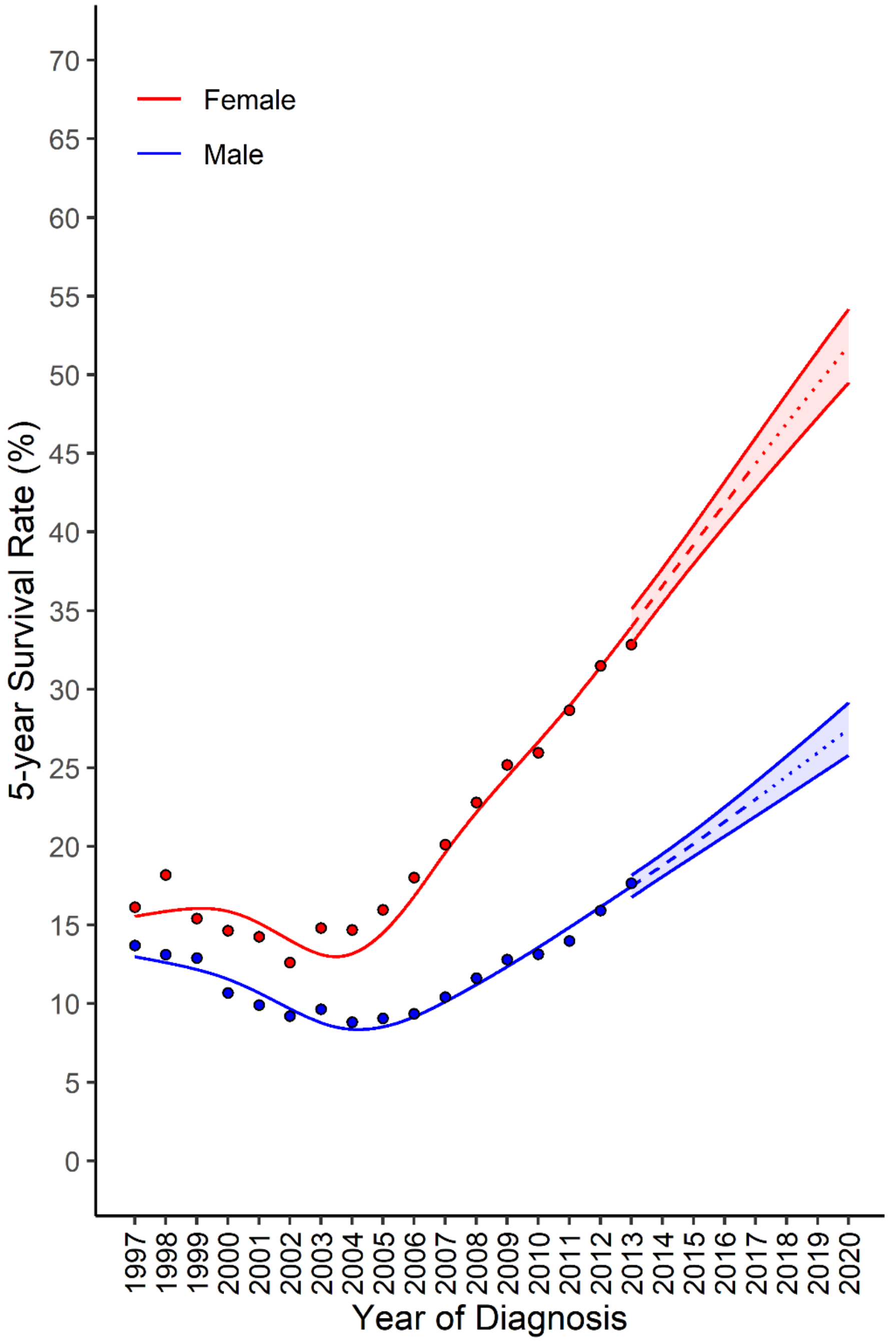

Figure S6. The 1-year interval survival rates (%) for lung cancer patients under 55 years old in Taiwan (dots: observed values; solid lines: fitted values from the survivorship-period-cohort model; dotted lines: projected values from the survivorship-period-cohort model; color-shaded regions: 95% bootstrap confidence intervals).

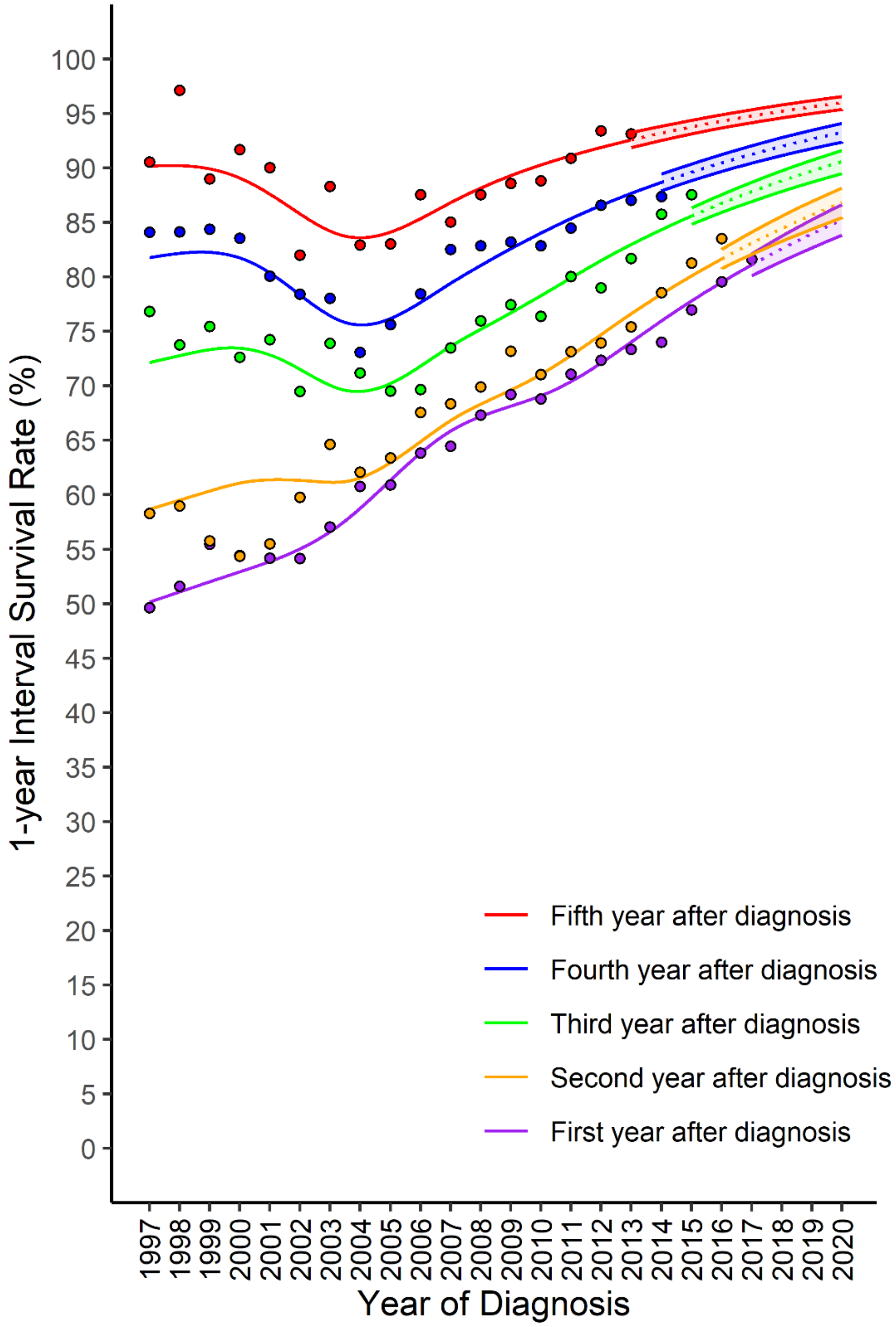

Figure S7. The 1-year interval survival rates (%) for lung cancer patients aged 55 to 64 years in Taiwan (dots: observed values; solid lines: fitted values from the survivorship-period-cohort model; dotted lines: projected values from the survivorship-period-cohort model; color-shaded regions: 95% bootstrap confidence intervals).

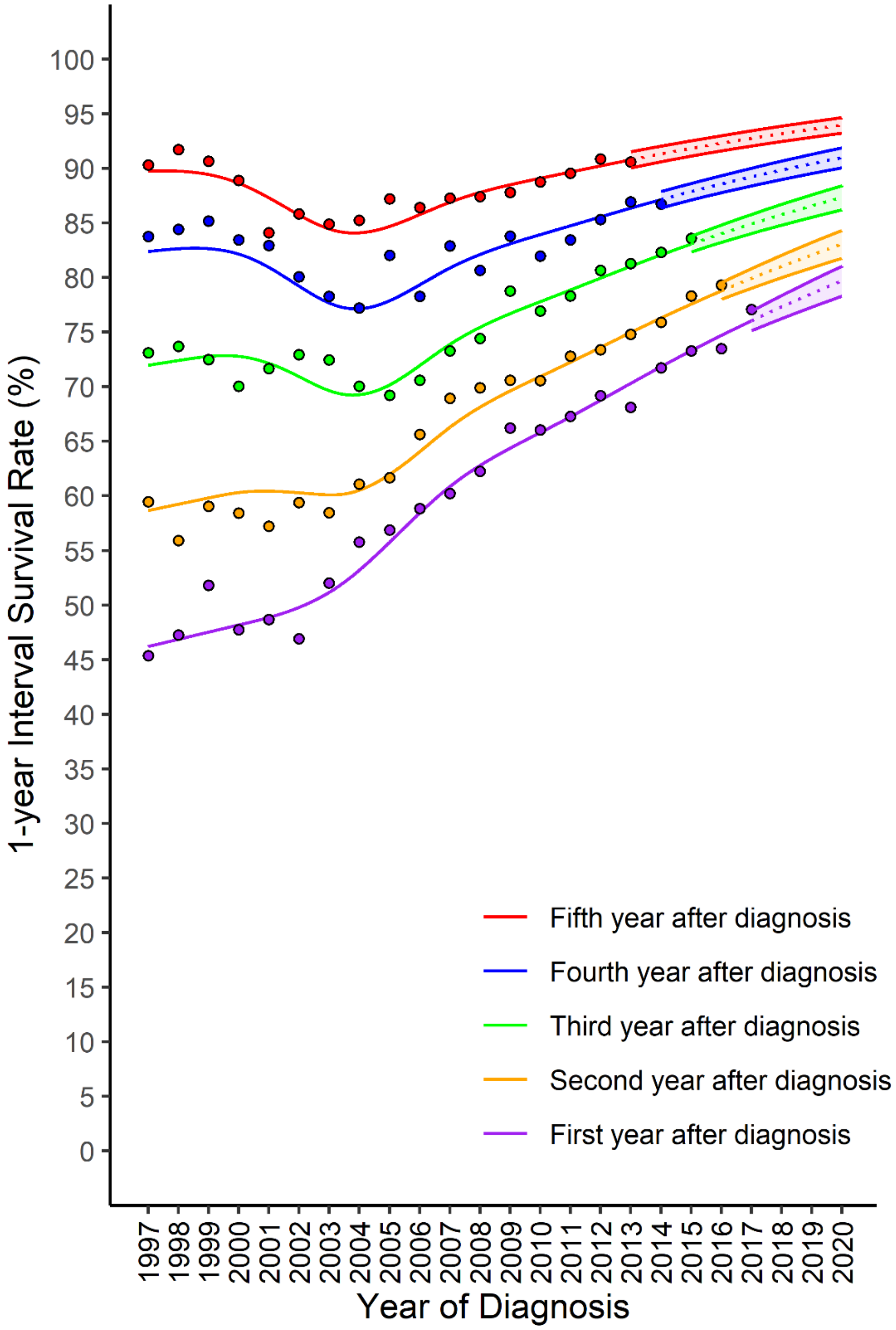

Figure S8. The 1-year interval survival rates (%) for lung cancer patients aged 65 to 74 years in Taiwan (dots: observed values; solid lines: fitted values from the survivorship-period-cohort model; dotted lines: projected values from the survivorship-period-cohort model; color-shaded regions: 95% bootstrap confidence intervals).

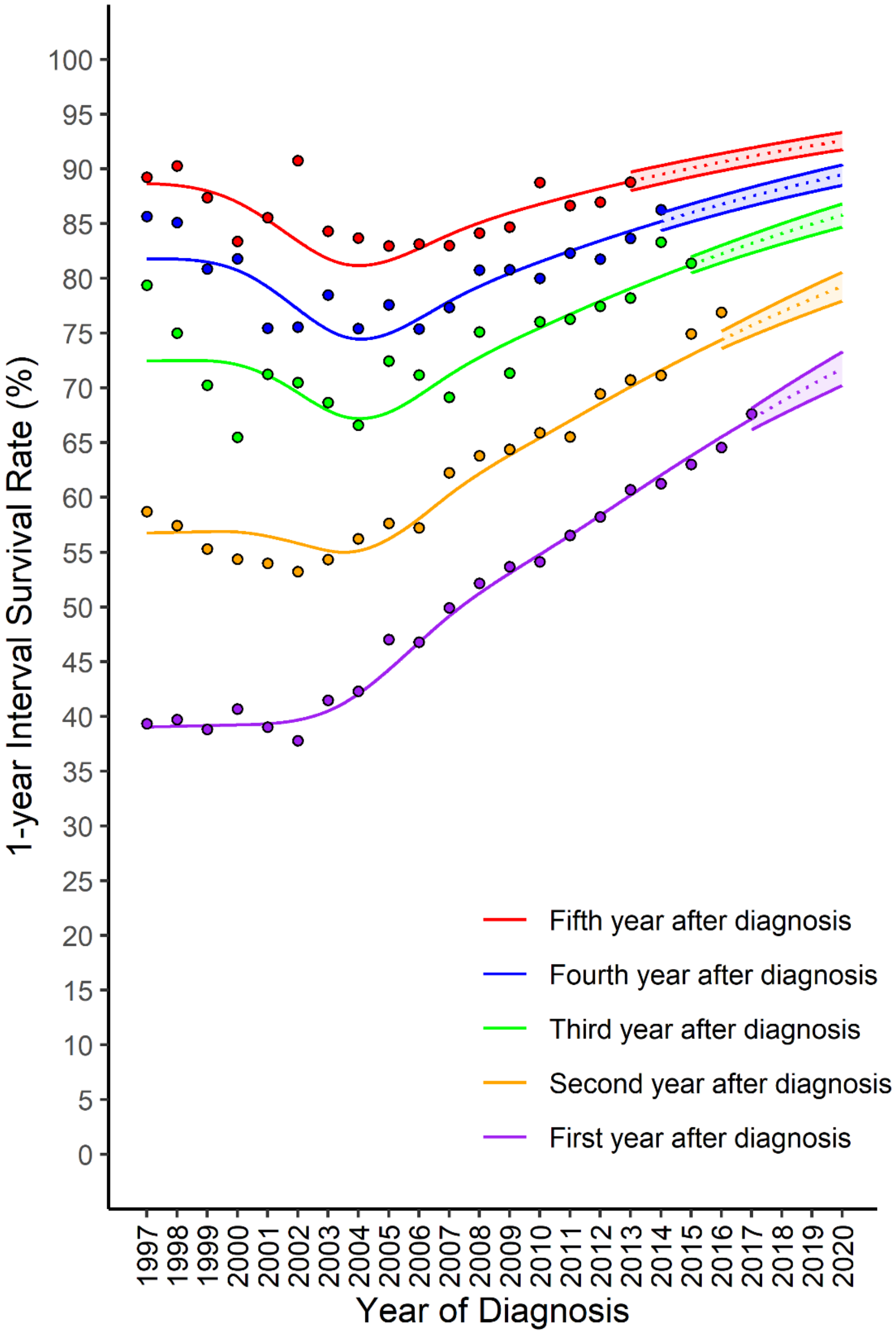

Figure S9. The 1-year interval survival rates (%) for lung cancer patients over 75 years old in Taiwan (dots: observed values; solid lines: fitted values from the survivorship-period-cohort model; dotted lines: projected values from the survivorship-period-cohort model; color-shaded regions: 95% bootstrap confidence intervals).

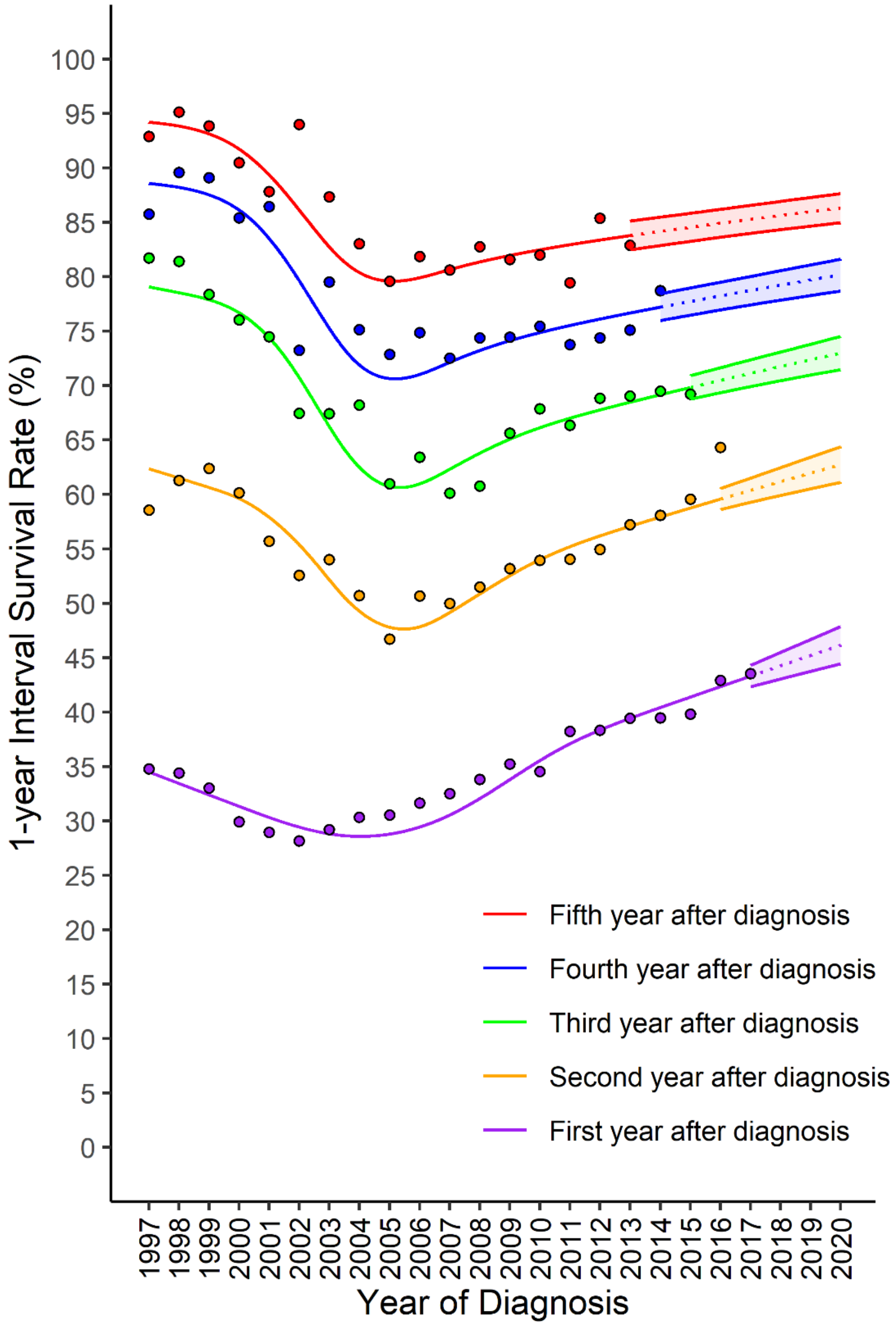

Figure S10. The 5-year survival rates (%) of lung cancer by age in Taiwan (dots: observed values; solid line: product of five observed 1-year interval survival rates from the first to fifth years after diagnosis for patients in the same diagnosis year, 1997-2013; dashed line: product of five partially observed and partially predicted 1-year interval survival rates from the first to fifth years after diagnosis for patients in the same diagnosis year, 2014-2017; dotted line: product of five predicted 1-year survival rates from the first to fifth years after diagnosis for patients in the same diagnosis year, 2018 to 2020; shaded region: 95% bootstrap confidence interval).

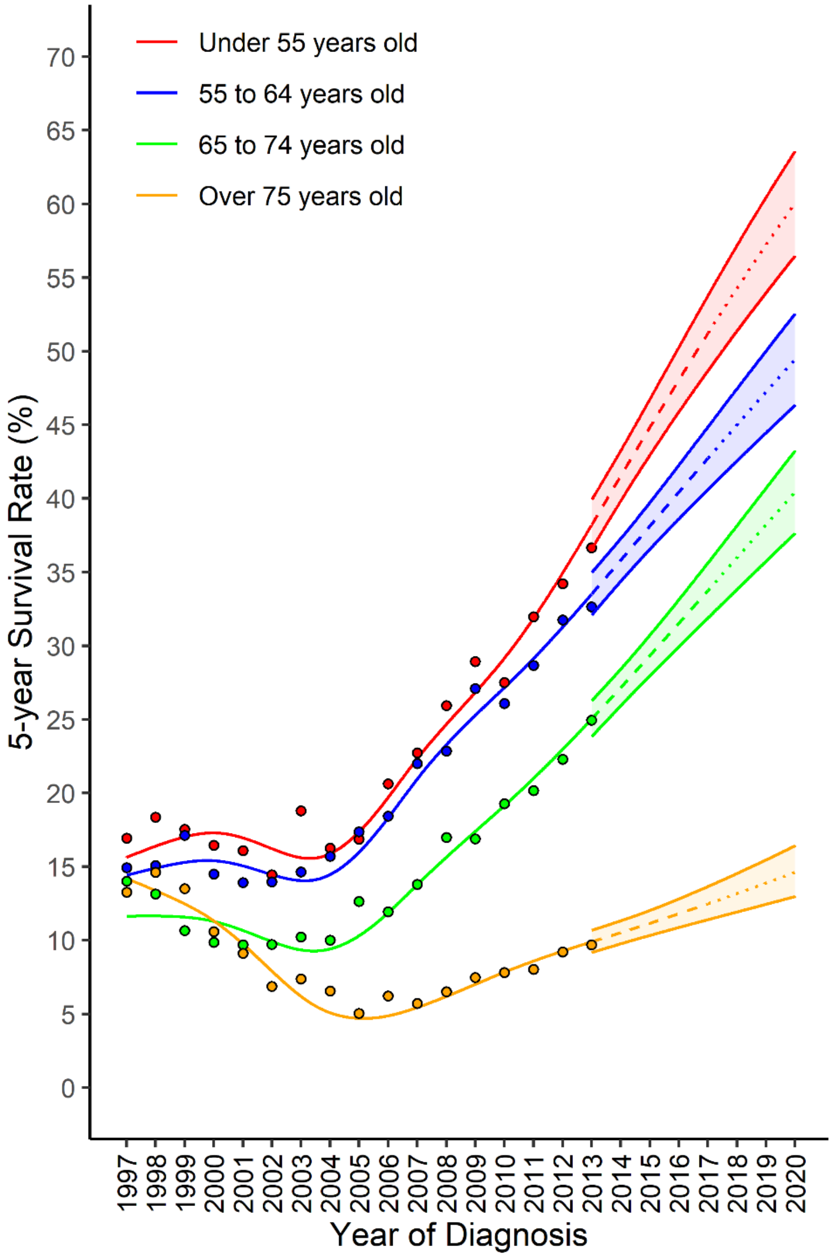

Figure S11. The 1-year interval survival rates (%) for lung adenocarcinoma in Taiwan (dots: observed values; solid lines: fitted values from the survivorship-period-cohort model; dotted lines: projected values from the survivorship-period-cohort model; color-shaded regions: 95% bootstrap confidence intervals).

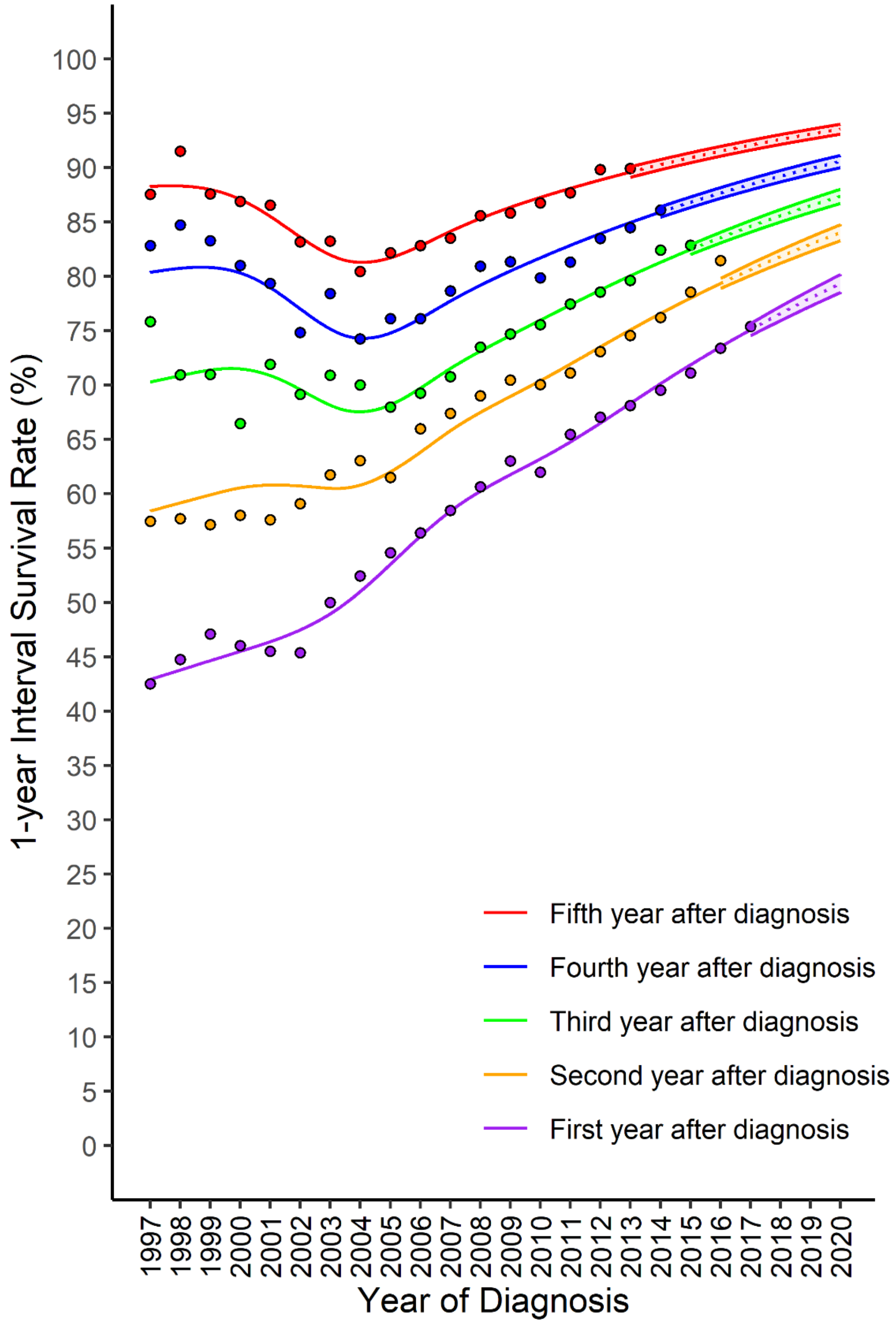

Figure S12. The 1-year interval survival rates (%) for lung squamous cell carcinoma in Taiwan (dots: observed values; solid lines: fitted values from the survivorship-period-cohort model; dotted lines: projected values from the survivorship-period-cohort model; color-shaded regions: 95% bootstrap confidence intervals).

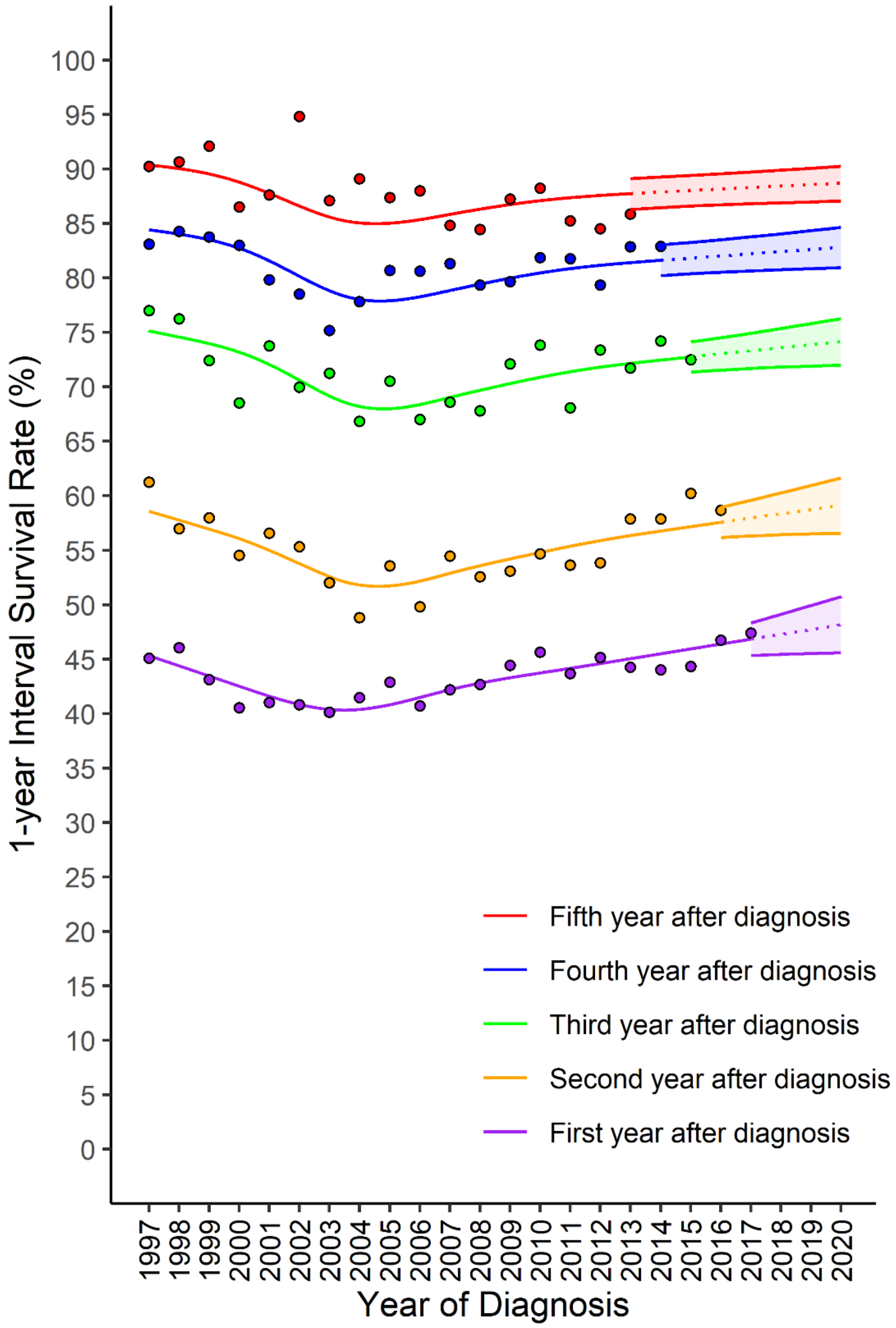

Figure S13. The 1-year interval survival rates (%) for small cell lung cancer in Taiwan (dots: observed values; solid lines: fitted values from the survivorship-period-cohort model; dotted lines: projected values from the survivorship-period-cohort model; color-shaded regions: 95% bootstrap confidence intervals).

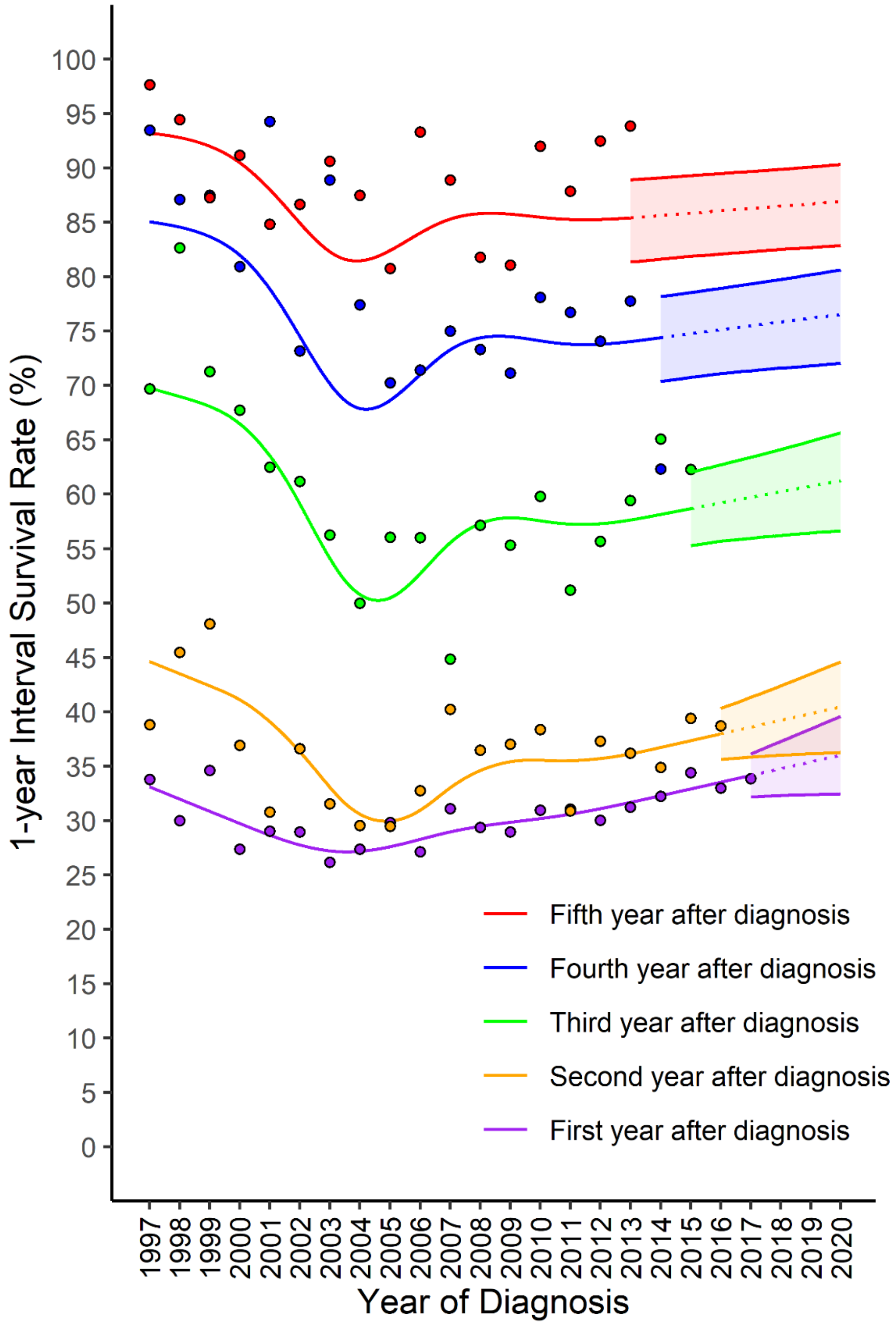

Figure S14. The 1-year interval survival rates (%) for other histological types of lung cancer in Taiwan (dots: observed values; solid lines: fitted values from the survivorship-period-cohort model; dotted lines: projected values from the survivorship-period-cohort model; color-shaded regions: 95% bootstrap confidence intervals).

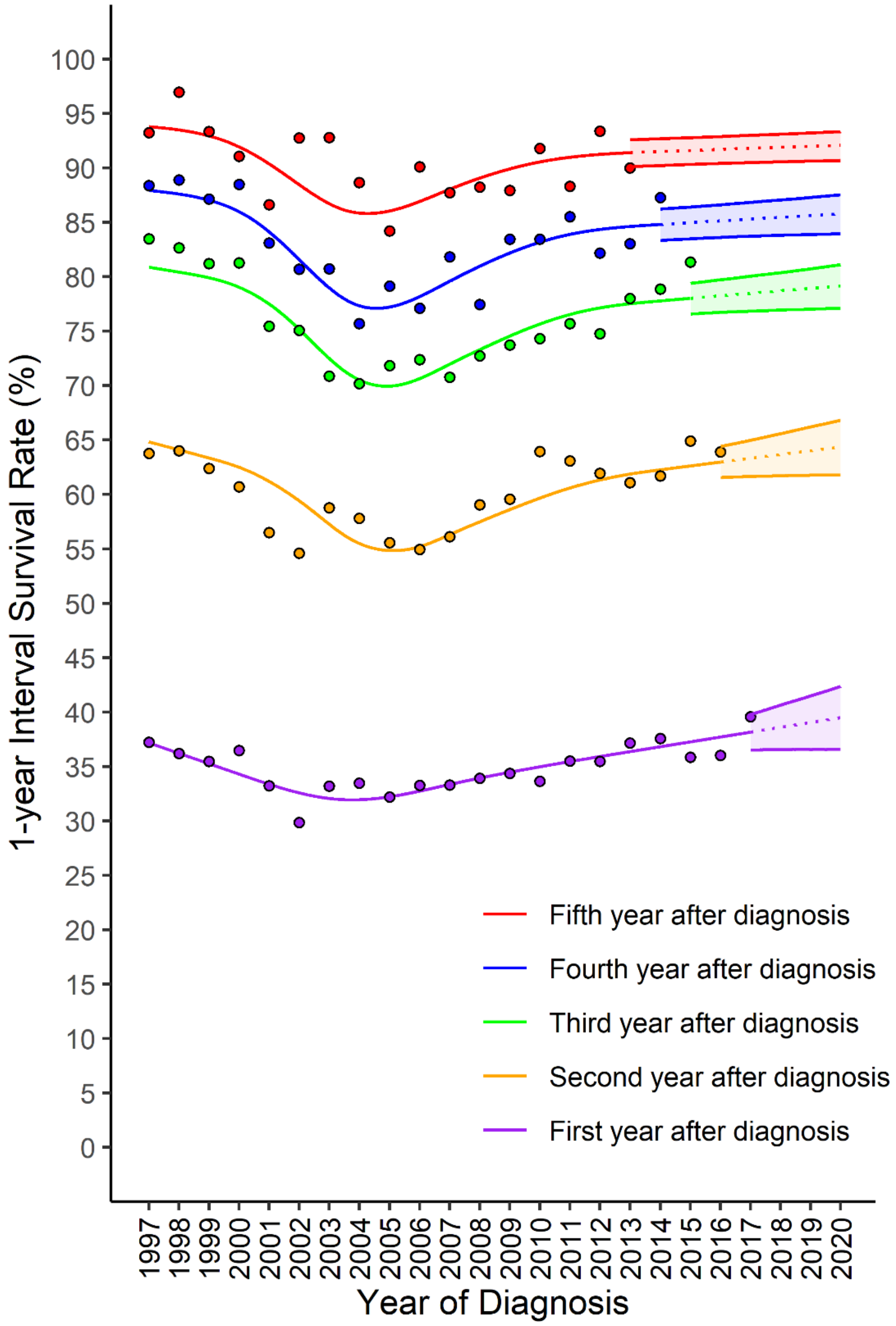

Figure S15. The 5-year survival rates (%) of lung cancer by histological type in Taiwan (dots: observed values; solid line: product of five observed 1-year interval survival rates from the first to fifth years after diagnosis for patients in the same diagnosis year, 1997-2013; dashed line: product of five partially observed and partially predicted 1-year interval survival rates from the first to fifth years after diagnosis for patients in the same diagnosis year, 2014-2017; dotted line: product of five predicted 1-year survival rates from the first to fifth years after diagnosis for patients in the same diagnosis year, 2018 to 2020; shaded region: 95% bootstrap confidence interval).

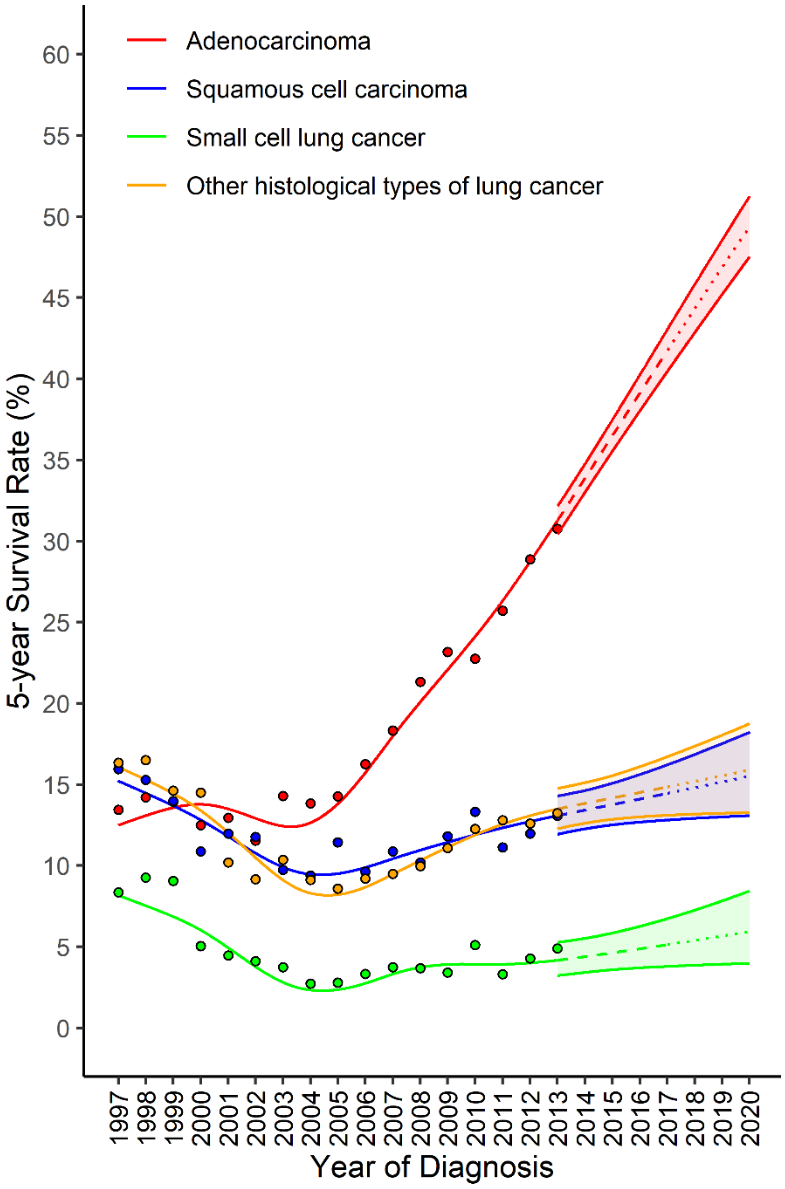

Figure S16. The proportion of lung cancer cases by stage in Taiwan (blue: stage I; orange: stage II; gray: stage III; yellow: stage IV).

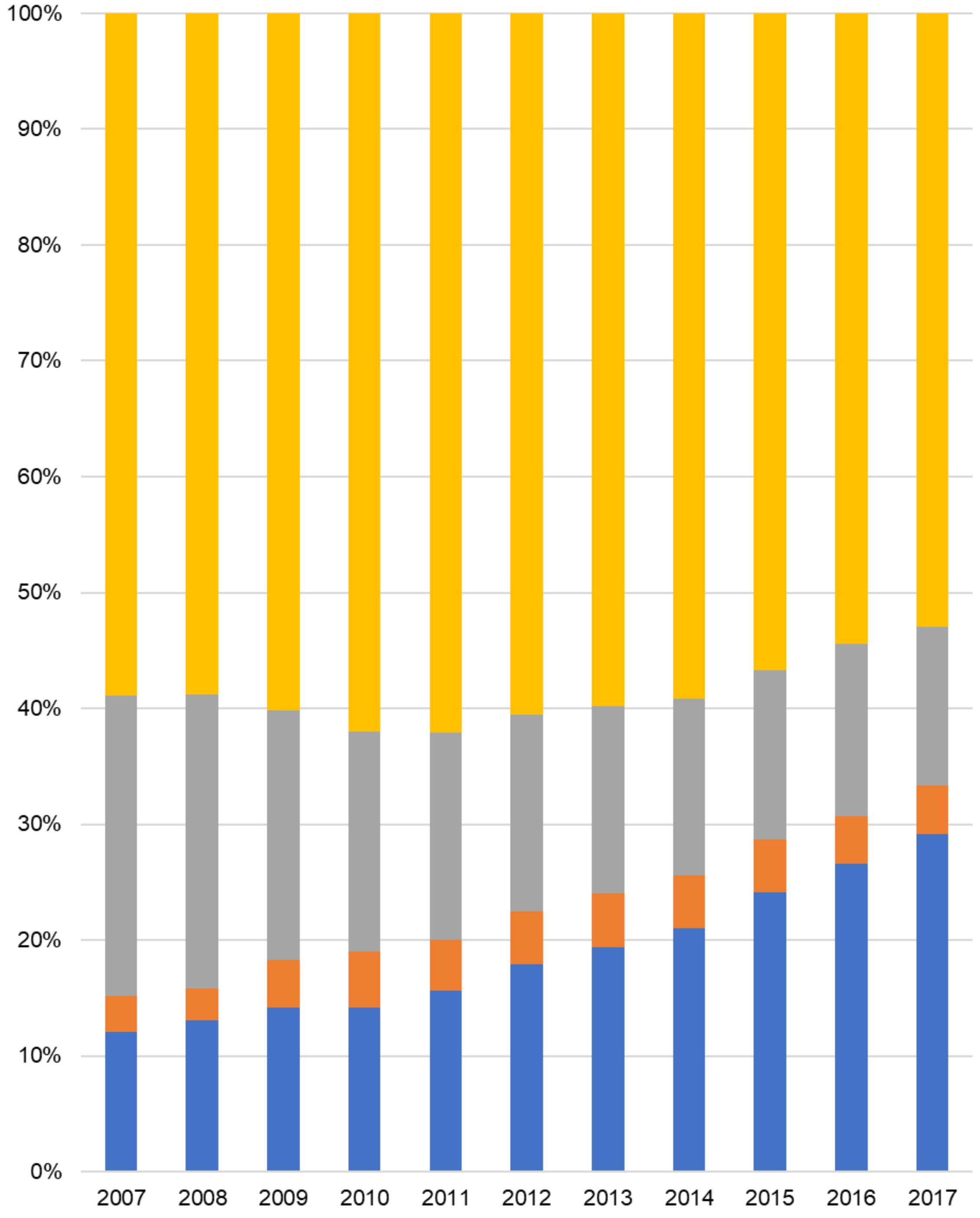

Figure S17. The combined 1-year interval survival rates (%) from stages I to IV of lung cancer in Taiwan. (dots: observed values; solid lines: fitted values from the survivorship-period-cohort model; dotted lines: projected values from the survivorship-period-cohort model; dot-dashed lines: 1-year interval survival rates (%) weighted average by the number of cases diagnosed at each stage in 2007 as weight; purple: first year after diagnosis; orange: second year after diagnosis; green: third year after diagnosis; blue: fourth year after diagnosis; red: fifth year after diagnosis; color-shaded regions: 95% bootstrap confidence intervals).

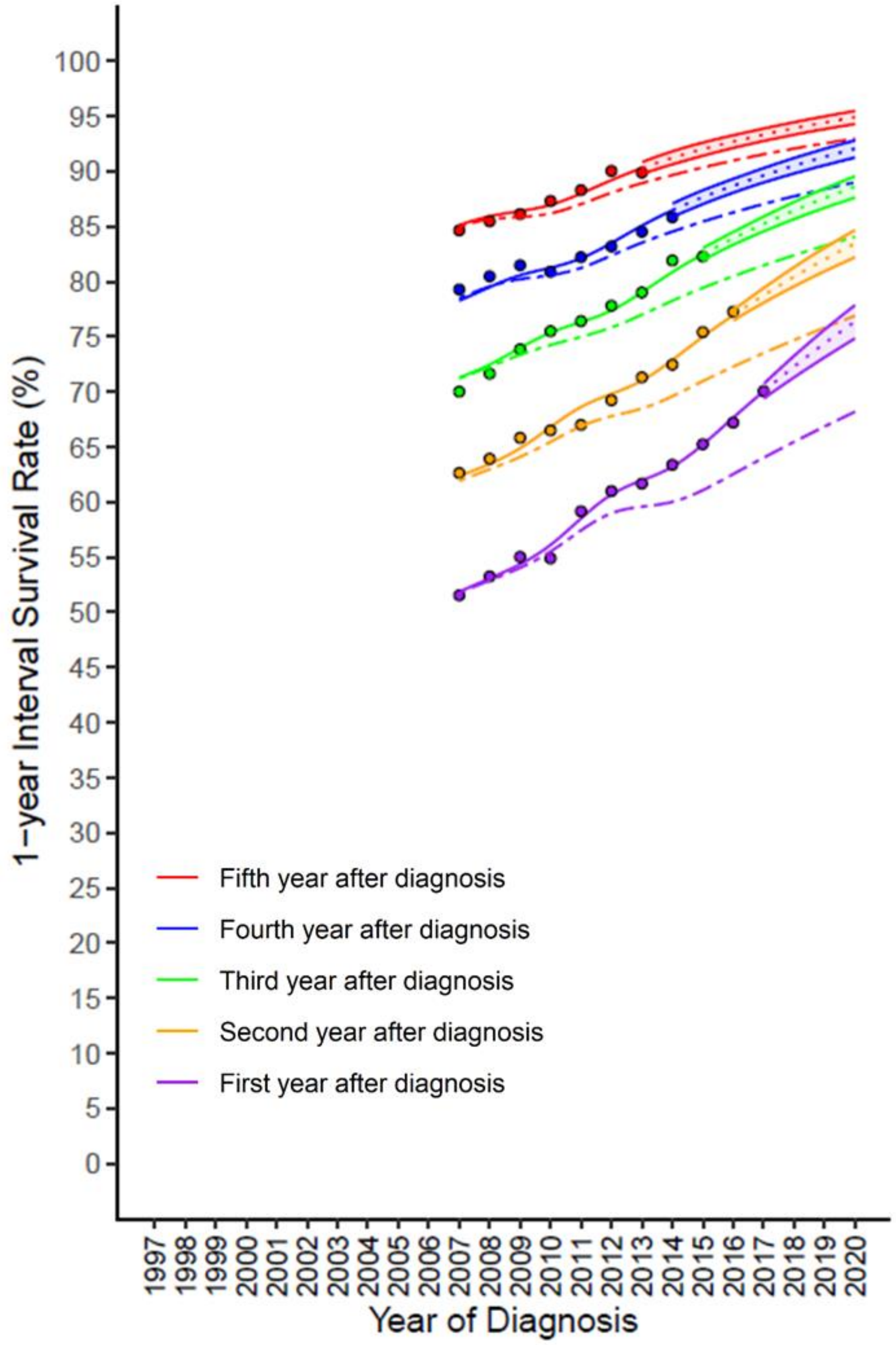

Figure S18. The combined 5-year survival rates (%) from stages I to IV of lung cancer in Taiwan. (dots: observed values; solid line: product of five observed 1-year interval survival rates from the first to fifth years after diagnosis for patients in the same diagnosis year, 2007-2013; dashed line: product of five partially observed and partially predicted 1-year interval survival rates from the first to fifth years after diagnosis for patients in the same diagnosis year, 2014-2017; dotted line: product of five predicted 1-year survival rates from the first to fifth years after diagnosis for patients in the same diagnosis year, 2018 to 2020; dot-dashed lines: 5-year survival rate (%) weighted average by the number of cases diagnosed at each stage in 2007 as weight.)

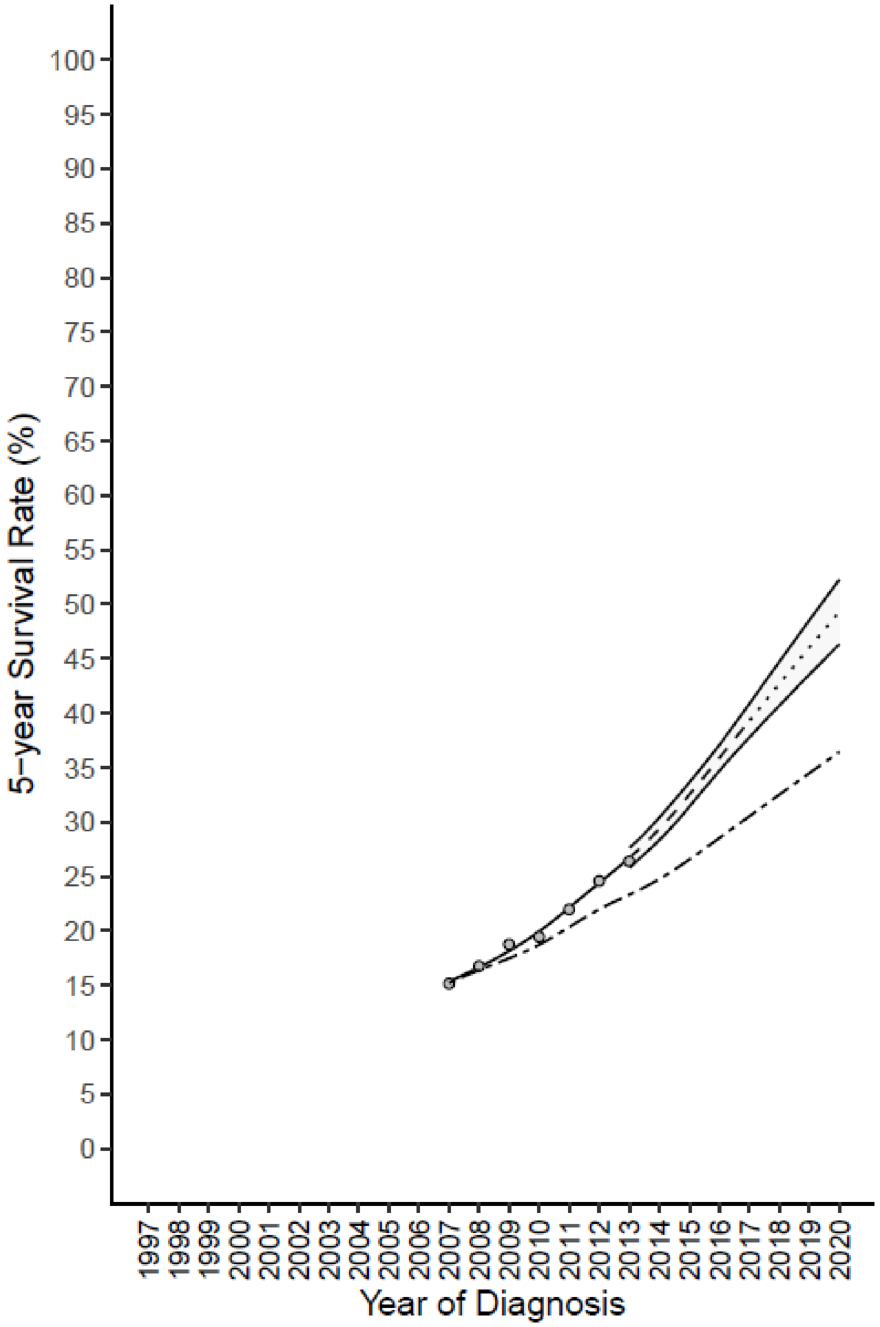

Figure S19. The 1-year interval survival rates (%) of stage I lung cancer in Taiwan (dots: observed values; solid lines: fitted values from the survivorship-period-cohort model; dotted lines: projected values from the survivorship-period-cohort model; color-shaded regions: 95% bootstrap confidence intervals).

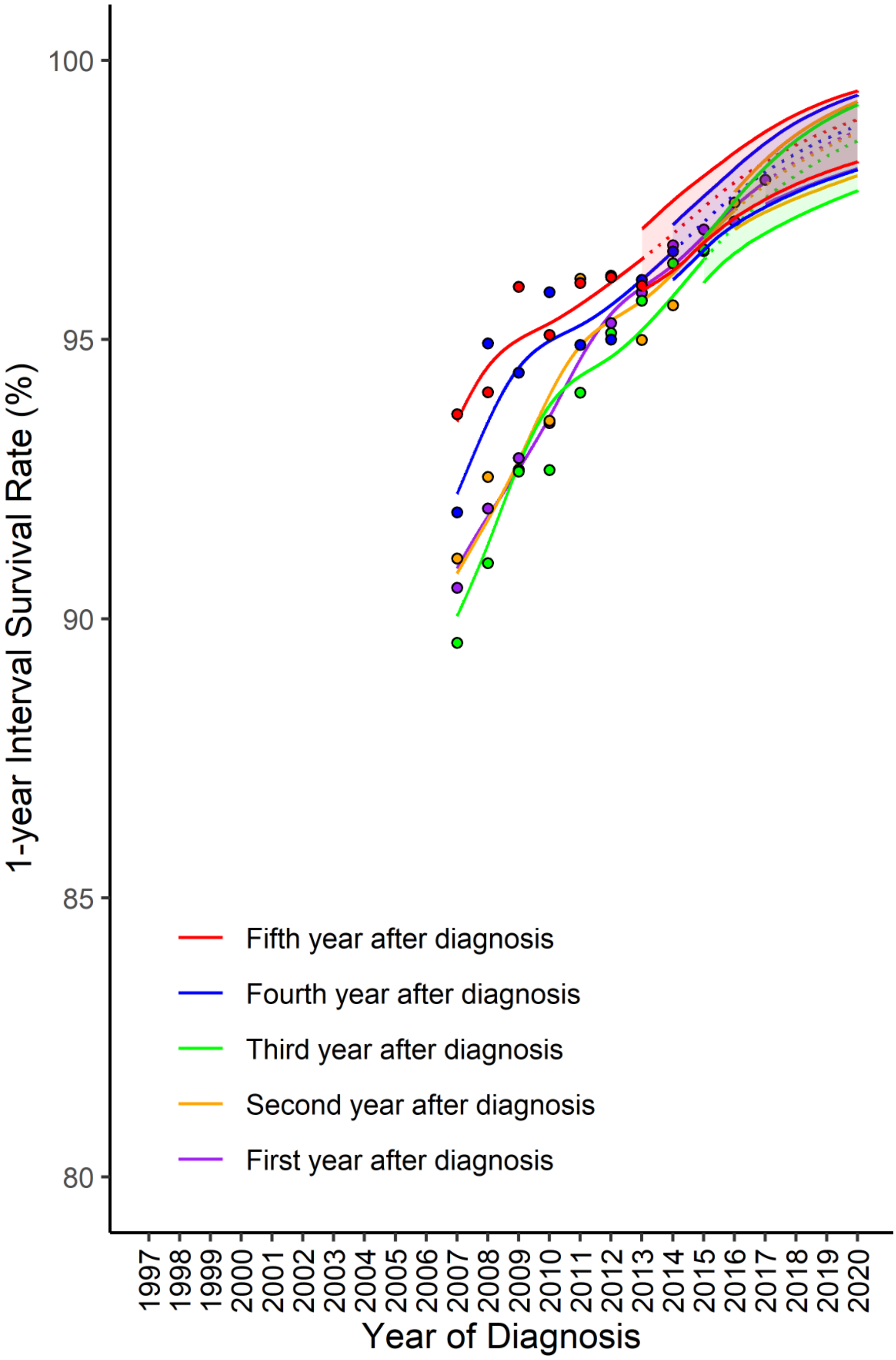

Figure S20.The 1-year interval survival rates (%) of stage II lung cancer in Taiwan (dots: observed values; solid lines: fitted values from the survivorship-period-cohort model; dotted lines: projected values from the survivorship-period-cohort model; color-shaded regions: 95% bootstrap confidence intervals).

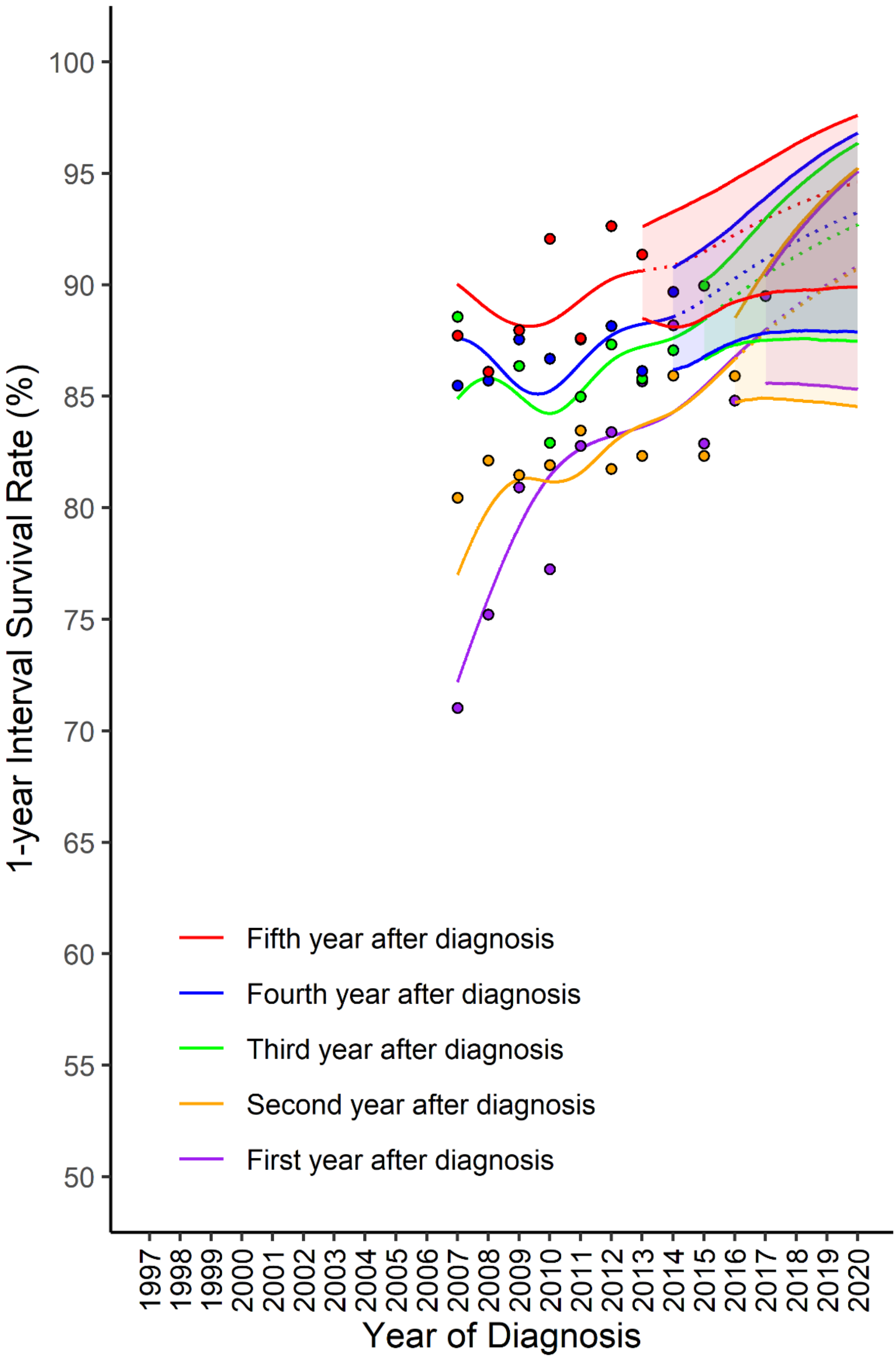

Figure S21. The 1-year interval survival rates (%) of stage III lung cancer in Taiwan (dots: observed values; solid lines: fitted values from the survivorship-period-cohort model; dotted lines: projected values from the survivorship-period-cohort model; color-shaded regions: 95% bootstrap confidence intervals).

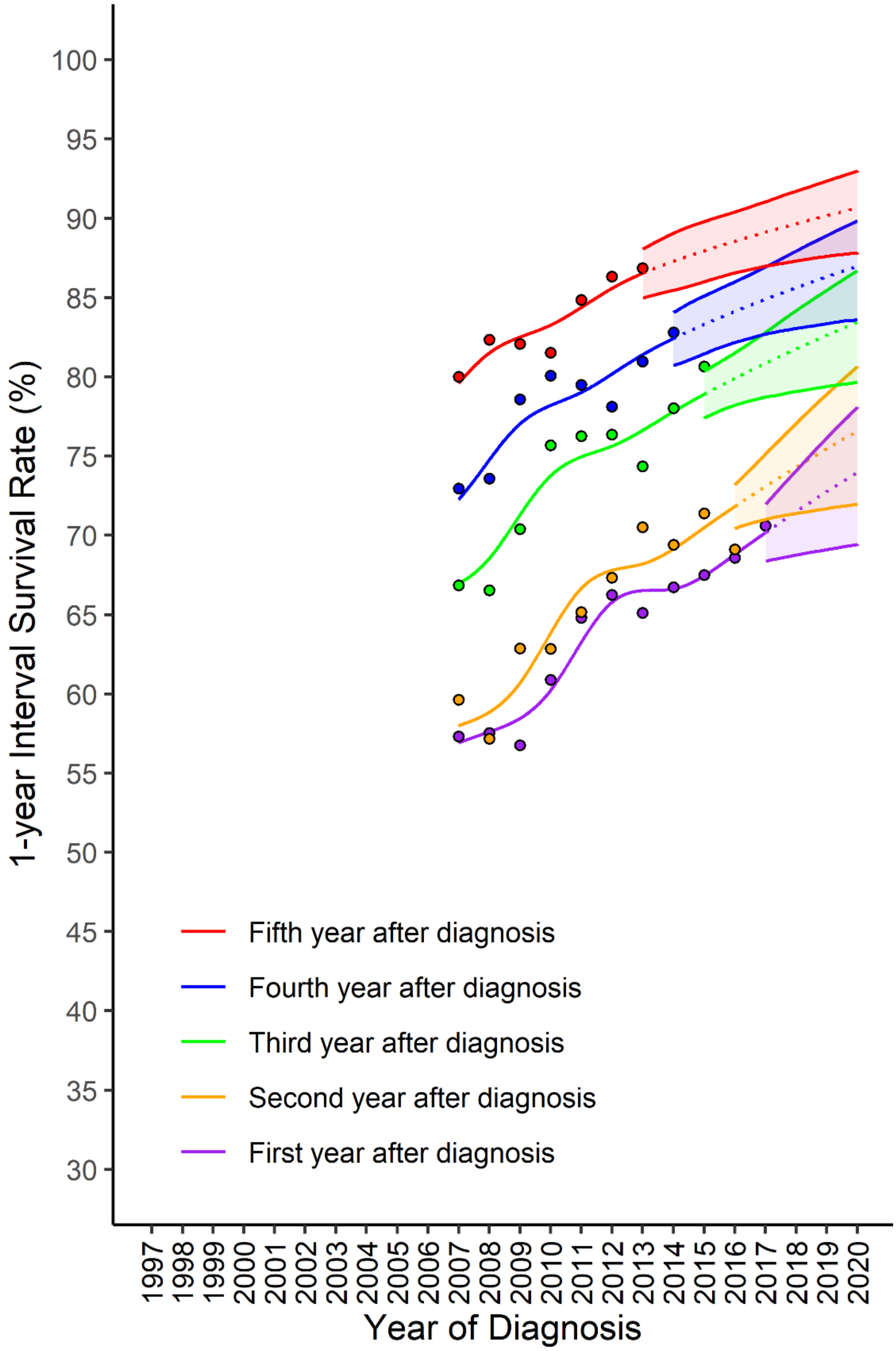

Figure S22. The 1-year interval survival rates (%) of stage IV lung cancer in Taiwan (dots: observed values; solid lines: fitted values from the survivorship-period-cohort model; dotted lines: projected values from the survivorship-period-cohort model; color-shaded regions: 95% bootstrap confidence intervals).

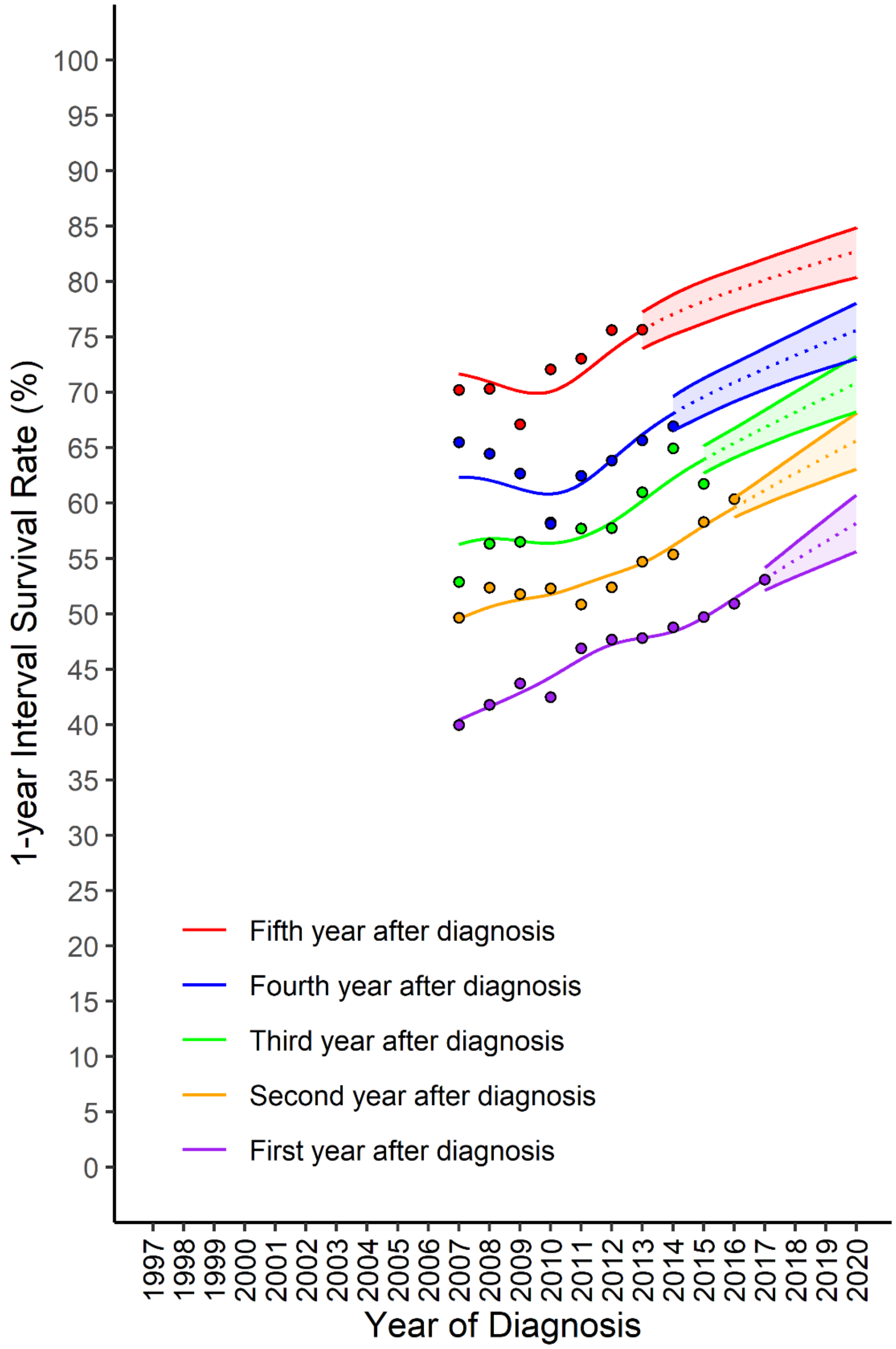

Figure S23. The 5-year survival rates (%) of lung cancer by stage in Taiwan (dots: observed values; solid line: product of five observed 1-year interval survival rates from the first to fifth years after diagnosis for patients in the same diagnosis year, 2007-2013; dashed line: product of five partially observed and partially predicted 1-year interval survival rates from the first to fifth years after diagnosis for patients in the same diagnosis year, 2014-2017; dotted line: product of five predicted 1-year survival rates from the first to fifth years after diagnosis for patients in the same diagnosis year, 2018 to 2020; shaded region: 95% bootstrap confidence interval).

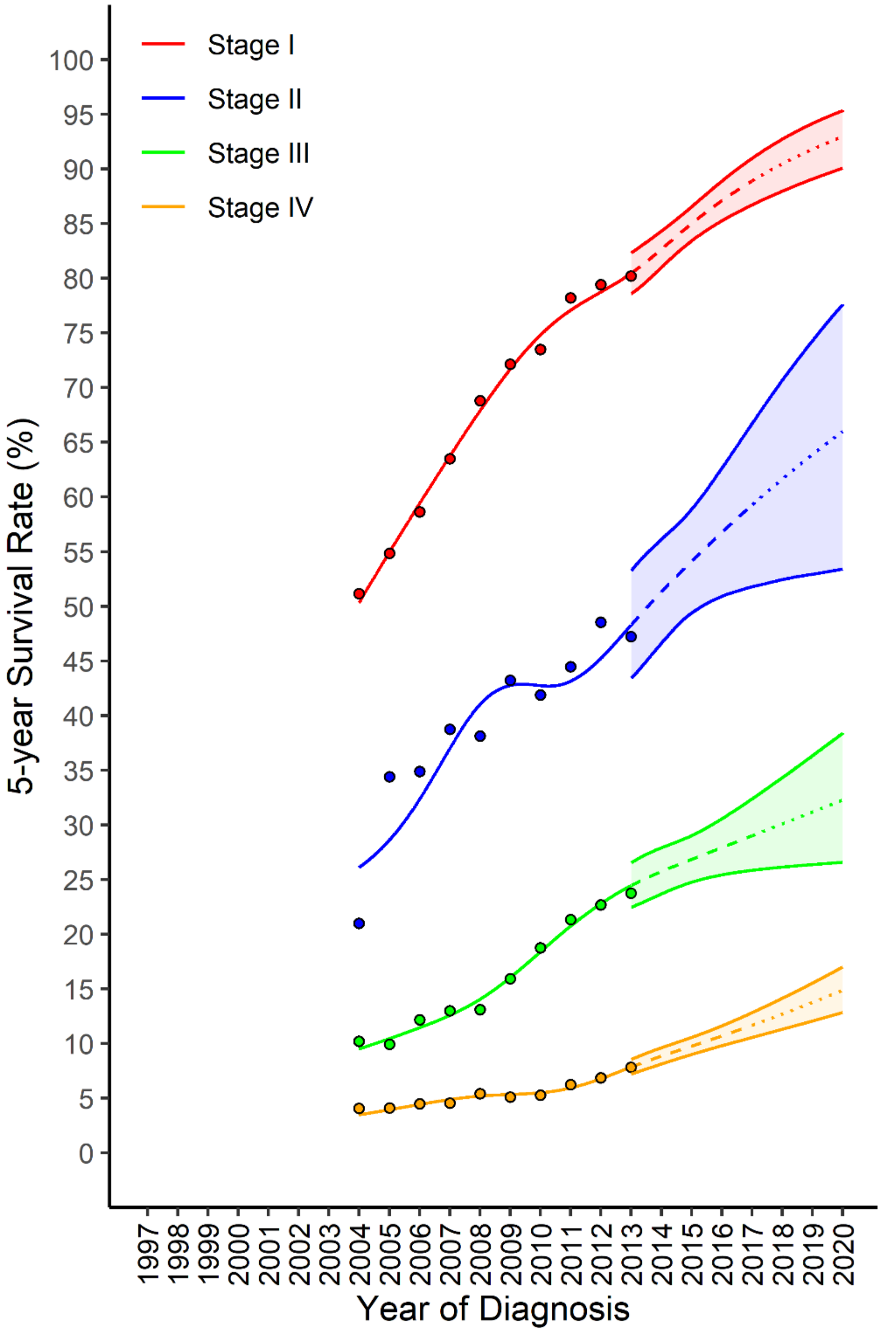

Supplement: Multimedia Appendix 1 [file publichealth_v10i1e46737_app1.pdf]
